# Bicc1 and Dicer regulate left-right patterning through post-transcriptional control of the Nodal inhibitor Dand5

**DOI:** 10.1038/s41467-021-25464-z

**Published:** 2021-09-16

**Authors:** Markus Maerker, Maike Getwan, Megan E. Dowdle, Jason C. McSheene, Vanessa Gonzalez, José L. Pelliccia, Danielle S. Hamilton, Valeria Yartseva, Charles Vejnar, Melanie Tingler, Katsura Minegishi, Philipp Vick, Antonio J. Giraldez, Hiroshi Hamada, Rebecca D. Burdine, Michael D. Sheets, Martin Blum, Axel Schweickert

**Affiliations:** 1 University of Hohenheim, Institute of Biology, Department of Zoology, Stuttgart, Germany; 2 Department of Biomolecular Chemistry, University of Wisconsin, Madison, WI USA; 3 Department of Molecular Biology, Princeton University, Princeton, NJ USA; 4 Department of Genetics, Yale University School of Medicine, New Haven, CT USA; 5 Laboratory for Organismal Patterning, RIKEN Center for Biosystems Dynamics Research, Hyogo, Japan; 6 Present Address: University of Zurich, Institute of Anatomy, Zurich, Switzerland

**Keywords:** Cell signalling, Embryonic induction, Embryonic induction, RNA decay

## Abstract

Rotating cilia at the vertebrate left-right organizer (LRO) generate an asymmetric leftward flow, which is sensed by cells at the left LRO margin. Ciliary activity of the calcium channel Pkd2 is crucial for flow sensing. How this flow signal is further processed and relayed to the laterality-determining Nodal cascade in the left lateral plate mesoderm (LPM) is largely unknown. We previously showed that flow down-regulates mRNA expression of the Nodal inhibitor Dand5 in left sensory cells. De-repression of the co-expressed Nodal, complexed with the TGFß growth factor Gdf3, drives LPM Nodal cascade induction. Here, we show that post-transcriptional repression of *dand5* is a central process in symmetry breaking of *Xenopus*, zebrafish and mouse. The RNA binding protein Bicc1 was identified as a post-transcriptional regulator of *dand5* and *gdf3* via their 3′-UTRs. Two distinct Bicc1 functions on *dand5* mRNA were observed at pre- and post-flow stages, affecting mRNA stability or flow induced translational inhibition, respectively. To repress *dand5*, Bicc1 co-operates with Dicer1, placing both proteins in the process of flow sensing. Intriguingly, Bicc1 mediated translational repression of a *dand5* 3′-UTR mRNA reporter was responsive to *pkd2*, suggesting that a flow induced Pkd2 signal triggers Bicc1 mediated *dand5* inhibition during symmetry breakage.

## Abstract (web summary)

The authors show that post-transcriptional regulation of the cilia-driven leftward flow target *dand5* is central to symmetry breakage in frog, fish and mouse and is mediated by a 139 nt Bicc1 responsive element in the *dand5* 3′UTR, and they present evidence that Pkd2 regulates this Bicc1/*dand5* module.

## Introduction

The Nodal signaling cascade is central in setting up organ situs during embryonic development^1,2^. In *Xenopus*, the Tgfβ ligand Nodal1 is activated in the LPM of the neurula embryo, where it induces its own transcription, that of its feedback inhibitor *lefty* and the homeobox transcription factor *pitx2*. Cilia are required for Nodal cascade induction^3–5^ in fish, amphibian and mammalian embryos, but not in reptiles and birds^1,6,7^. The archenteron (primitive gut or remnants thereof) transiently harbors the ciliated epithelium of the left-right organizer (LRO) during neurula stages. In frogs, the gastrocoel roof plate (GRP^8^) develops from precursor cells, i.e., superficial mesoderm, which is specified during the early gastrula stages. GRP cells have different fates, which correlate with specific properties. They are notochordal (cLRO) at the midline or somitic (sLRO) more laterally. The GRP, like other vertebrate LROs, is typically characterized by motile cilia at its center and immotile, supposedly sensory cilia at its lateral borders^5^. The posterior orientation and tilt of motile cilia, and their intrinsic clockwise rotation, give rise to a leftward fluid flow in the extracellular space that is sensed at the left LRO margin by a ciliary complex containing the ion-channel Pkd2 (TRPP2/Polycystin2). It is generally believed that the cation channel Pkd2, which we initially characterized as an LR determinant in a *pkd2* knockout mouse^9^, is central to flow sensing. In mice and fish, left-asymmetric calcium spikes in lateral LRO cells were reported, which were depending on a ciliary Pkd2 function. Calcium influx, therefore, seems to represent the initial response to flow sensing^10,11^.

The decisive molecular target of leftward flow is the repression of the Nodal inhibitor *dand5* (former *coco* in frog; *Cerl2* in mouse; *charon* in fish) at the left LRO margin^12–14^. *nodal* is co-expressed with *dand5* in time and/or space and thereby inhibition of Dand5 protein synthesis results in de-repression of Nodal signaling. As a consequence, Nodal bound to the Gdf3 protein (former *derriére* in frog; *Gdf1* in mouse) is transferred to the left LPM, where it induces the left-asymmetric Nodal signaling cascade^15,16^. A critical component of LR patterning is the flow-dependent repression of Dand5, manifested partially by a left-sided reduction of *dand5* mRNA in vertebrate embryos. In mice, *dand5* mRNA is destabilized via its 3′-UTR in a flow-dependent manner^13^. However, the timing of *dand5* asymmetry raises the possibility that post-transcriptional mRNA decay might be insufficient for reducing Dand5 protein levels and suggests that additional mechanisms contribute to repression. In frog, *dand5* mRNA asymmetry is most pronounced at late neurula, i.e., the very stages (st. 19–21) in which *nodal1* is already expressed in the left LPM^12^. In addition, left-sided *dand5* mRNA decay in *Xenopus* is observed in a maximum of ~80% of wt specimens, whereas left Nodal cascade induction and the arrangement of inner organs were undisturbed in 95% of cases. Thus, the frequency of *dand5* asymmetry is insufficient to explain the robust occurrence of wildtype organ asymmetry (situs solitus). The data indicates that detectable *dand5* mRNA asymmetry occurs too late and infrequently to be functionally relevant and suggests that flow-dependent *dand5* repression might also include translational inhibition.

A protein that could exert both proposed post-transcriptional functions is Bicc1 (BicC family RNA-binding protein 1). Bicc1 binds to selected mRNAs and modifies translation post-transcriptionally, in a positive^17^ or negative context-dependent manner^18,19^. Further, Bicc1 localizes to P-bodies, cytoplasmic complexes involved in mRNA stability and turnover^17,20,21^. Interestingly, Bicc1 interacts with microRNAs (miRs), which function in post-transcriptional regulation of mRNA translation and integrity^22^. Previous studies indicated Bicc1 functions are important for LR patterning. *bicc1* in frogs and mice is expressed in the LRO. *bicc1* loss-of-function (LoF) impacts Wnt/planar cell polarity (PCP) signaling, resulting in unpolarized LRO cilia and perturbed leftward flow^20^. However, *bicc1* expression in the frog LRO revealed strong enrichment of mRNA in *nodal1* and *dand5* positive sLRO cells (cf. Figure 3 in ref. ^20^), indicative of a separate, specific function in these cells, which was not addressed at the time.

Here, we show that the RNA-binding protein Bicc1 regulates *dand5* mRNA stability and translation in LRO sensor cells. Approximately 139 nucleotides of the proximal *dand5* 3′-UTR are required and sufficient for Bicc1-mediated translational repression. Furthermore, within this small  sequence, we identify distinct sub-regions specific for *dand5* mRNA stability and translational repression. In addition, we show that Bicc1 also regulates the translation of *gdf3*, thereby influencing Nodal signaling directly. Finally, our data indicate that *bicc1* functions together with *dicer1* (the enzyme catalyzing the final step of miR biosynthesis) and *pkd2* to mediate *dand5* repression, and this function is evolutionary conserved in other organisms.

## Results

### Bicc1 represses *dand5* translation

Because of the allotetraploid genome of *Xenopus laevis*, 3′-UTRs of *dand5* alloalleles were compared. Sequence conservation between S and L-alleles is low, except for the proximal 230 nucleotides downstream of the Stop-codon, which show 84% sequence identity (Supplementary Fig. 1A, B). Asymmetric *dand5* mRNA expression was found for both alleles, as visualized by in situ hybridization (ISH) of dorsal explants at stages 18 and 20 with antisense RNA probes specific for the 3′-UTRs of S- and L-alleles (Supplementary Fig. 1C, D).

**Fig. 1:**
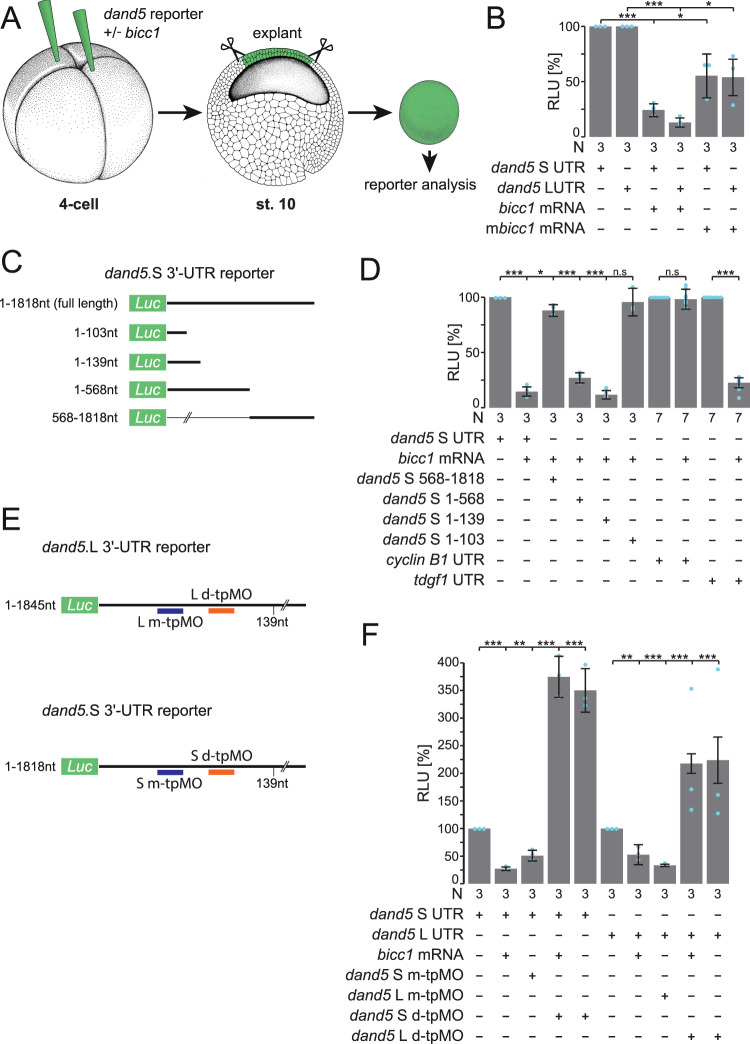
Bicc1 represses *dand5* mRNA translation via its proximal 3′-UTR. **A** Schematic depiction of *dand5* reporter assay. *dand5* 3′-UTR sequences fused to luciferase coding were injected either with or without *bicc1* mRNA into the animal region of four-cell embryos. At st. 10, the animal cap region was excised and assayed for luciferase activity. Adapted from refs. ^65^ and ^66^. **B** Animal cap reporter assay following injections of *dand5* S- or L 3′-UTRs alone or together with *Xenopus* (*bicc1*) or mouse *Bicc1* (*mbicc1*) effector mRNAs. Note that both alloalleles were equally repressed. Note also that *mbicc1* was efficient as a repressor as well. **C** Luciferase reporter constructs harboring different regions of the *dand5* (S-allele) 3′-UTR. **D** Repression of translation is mediated through a proximal 139 nucleotides (nt) sequence element in the *dand5* 3′-UTR. **E** Schematic depiction of medial and distal target protector MOs (m-tpMOs or d-tpMOs) binding to the minimal Bicc1 responsive element (Bicc1RE) in the *dand5* 3′-UTR (L or S). **F** m- and d-tpMOs (0.4 or 0.5 pmol/embryo, respectively) interact differently with the luciferase reporter expression. m-tpMO blocked and d-tpMO boosted luciferase activity. Co-injection of d-tpMOs prevented Bicc1-dependent repression of the full-length *dand5* reporters (L and S) and further enhanced their expressivity. N in **B**, **D**, and **F** represents the number of independent experiments. A pool of 10 animal caps was analyzed per experiment and treatment. Results from reporter mRNAs alone served as reference and were set to 100% RLU. Relative values of single experiments are depicted as blue dots. Data of at least three experiments are presented as mean value (bar) ±standard deviation (error bar, SD). Statistical analyses were done with a one-sided Student’s *t* test for two independent means (Bonferroni corrected) using the values of at least three individual experiments. *p* values, values for individual experiments, the mean values, and standard deviations are found in the source data file. n.s. not significant *p* > 0.05; * significant, *p* < 0.05; ** highly significant *p* < 0.01; ***, very highly significant *p* < 0.001; RLU relative luciferase units; *Luc* *luciferase*.

Based on our previous identification of *dand5* mRNA as a target of Bicc1 binding^18^ we wondered whether Bicc1 regulates *dand5*, the critical effector downstream of leftward flow^1^. To directly test this, an assay in animal cap explants was set up (AC assay; Fig. 1A) which specifically analyzed Bicc1’s capacity to interfere with mRNA translation. We used mRNAs of our previously published *dand5*.S 3′-UTR and newly cloned L 3′-UTR luciferase reporters (Supplementary Fig. 1B^18^). Because *bicc1* is not present in AC cells^23^, gain-of-function (GoF) experiments do not unravel any in vivo functions but reflect a somewhat artificial assay. *dand5* mRNA, however, is maternally deposited at the animal pole^24^ and thus post-transcriptional regulation might be active in AC cells. Injections were targeted to the animal region of the four-cell embryo and AC tissue was excised at the early gastrula stage 10 (Fig. 1A). Reporter mRNAs harboring the full-length 3′-UTRs of the respective S and L-alleles of *dand5* were translated, subsequently resulting in luciferase activity (Fig. 1B). Co-injection of *bicc1* mRNA, however, repressed luciferase activity of both reporters to ~20%. A full-length mouse *bicc1* mRNA also repressed reporter activities, though less efficiently (Fig. 1B). These experiments demonstrate a repressive effect of Bicc1 on a reporter protein expressed from an mRNA construct harboring the *dand5* 3′-UTR.

Next, we asked which sequences in the 3′-UTR were required for translational inhibition by Bicc1. To do so, different regions of the 1818 nucleotides of *dand5*.S 3′-UTR were deleted (Fig. 1C). Deleting the proximal 568 nucleotides abrogated the repressing effect of Bicc1 (Fig. 1D). This proximal sequence alone enabled translational repression to ~30% of wt, i.e., slightly less than the full-length 3′-UTR. Further narrowing down to nucleotides 1–139 allowed repression at wt levels while deleting additional 26 nucleotides (construct 1–103) abolished translational inhibition, suggesting that nucleotides 103–139 could be particularly important. For validation, a *cyclin* B1 reporter was used as negative, and a *tdgf1* (*cripto*) reporter as a positive control, as previously reported^18^.

To test whether the proximal element of the *dand5* 3′-UTR was instrumental in mediating Bicc1-dependent translational repression, antisense target protector morpholino oligomers (tpMOs) were designed. tpMOs have been recently used to block specific sequences in UTRs, i.e., miRNA or protein-binding sites, to analyze post-transcriptional regulation^25^. We specifically targeted the distal 103–139 region (d-tpMO) and 5′ adjacent medial sequences (m-tpMO) of the identified minimal Bicc1 responsive 3′-UTR in S and L. The medial tpMOs were complementary to nucleotides 65–89 for the L and S alloalleles (*dand5*.L m-tpMO; *dand5*.S m-tpMO). The distal tpMOs were complementary to nucleotides 91–116 of the L (*dand5*.L d-tpMO) and 107–132 of the S (*dand5*.S d-tpMO) alloallele (Fig. 1E; Supplementary Fig. 1B). m-tpMOs alone efficiently repressed reporter mRNA translation, suggesting that the blocked sequence is critical for general expressivity (Fig. 1F). Co-injection of the d-tpMOs with the full-length 3′-UTR *dand5* reporter and *bicc1* mRNA prevented repression (Fig. 1F). The reporter activity was about two- to threefold enhanced by d-tpMOs, as was the reporter activity upon co-injection with d-tpMO in the absence of *bicc1* (Fig. 1F). These data show that additional components restrict *dand5* reporter activity through interaction with its 3′-UTR, suggesting that the endogenous *dand5* mRNA is under post-transcriptional control independently of Bicc1. Taken together the reporter assays confirm the role of the proximal 3′-UTR *dand5* sequences in Bicc1-dependent repression, which we, therefore, termed “Bicc1 responsive element” (Bicc1RE; Supplementary Fig. 1B).

### Bicc1 responsive element is required for LR asymmetry

**Fig. 2:**
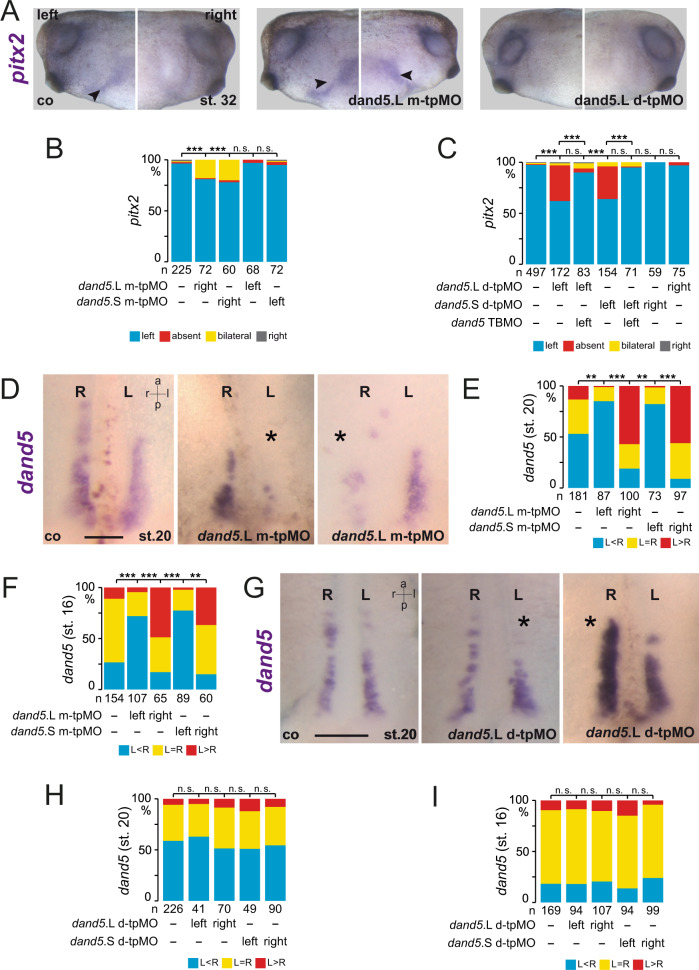
Bicc1 responsive element (Bicc1RE) of the *dand5* 3′-UTR is required for LR asymmetry. **A** Uninjected control (co), m-tpMO, or d-tpMO-injected embryos showed left, bilateral, or absent *pitx2* expression, respectively. Lateral views (left and right) of embryos are presented. Arrowheads mark *pitx2*-positive lateral plate mesoderm. **B** Quantification of *pitx2* results of m-tpMO-treated specimens. **C** Quantification of *pitx2* asymmetry by d-tpMO injections. Note administration of *dand5* TPMO together with d-tpMOs restored wt *pitx2* expression. **D** Diminished *dand5* mRNA expression by left-sided and right-sided m-tpMO injections compared with control. **E** Quantification of *dand5* expression at post-flow stages (st.20) following m-tpMO treatment. **F** Quantification of *dand5* expression in pre-flow specimens injected with m-tpMO. **G** Wildtype *dand5* repression in control (co) and left- or right-sided d-tpMO injected specimens. **H** Quantification of *dand5* asymmetry. Note flow-induced *dand5* mRNA decay was observed in controls and following d-tpMO application. **I** Quantification of *dand5* staining of pre-flow specimens (st.16) following d-tpMO injections. MO pmol/embryo: m-tpMO (L or S, 0.8); d-tpMO (L or S, 1). Asterisks in **D** and **G** mark injected side. Scale bars in **D** and **G** represent 100 µm. Numbers (n) in **B**, **C**, **E**, **F**, **H**, and **I** represent analyzed specimens from more than three independent experiments. Statistical analyses were done with one-sided Pearson’s chi-square test, which was adjusted for multiple comparisons by Bonferroni (**B**, **C**) or Bonferroni–Holm (**E**, **F**, **H**, **I**). *p* values and listing of individual experiments can be found in the source data file. n.s. not significant *p* > 0.05; **highly significant *p* < 0.01; ***very highly significant *p* < 0.001; st. stage; a anterior; l left; r right; p posterior.

To underscore the functional relevance of our AC assays, we examined the in vivo effect of tpMOs on LR asymmetry. Injections were performed in a unilateral manner at the 4–8 cell stage and thereby the effects on flow receiving, left or right sLRO cells were analyzed separately. Targeting m-tpMOs (L or S) to left sLRO cells did not change *pitx2* asymmetry at tadpole stages, whereas on the right, m-tpMOs induced ectopic *pitx2* expression in the right LPM (Fig. 2A, B). In contrast, right-sided injections of d-tpMOs (either L or S allele) had no effect, whereas left application prevented *pitx2* induction in the left LPM in close to 50% of specimens (Fig. 2A, C), suggesting that Bicc1RE is also required for *dand5* repression in vivo. Importantly, co-injection of *dand5* translation blocking morpholino (TBMO) rescued asymmetric *pitx2* induction (Fig. 2C), emphasizing d-tpMO specificity. These results suggest that medial and distal sub-regions of the Bicc1RE in the *dand5* 3′-UTR mediate different aspects of *dand5* post-transcriptional regulation and therefore both impact on LR asymmetry.

We then analyzed *dand5* expression patterns following tpMO treatment. *dand5* mRNA expression at post-flow stages (st. 20) was considerably reduced by m-tpMO irrespective of whether the right or left sLRO lineage was targeted (Fig. 2D, E). Sided *dand5* downregulation by m-tpMO was in agreement with either ectopic *pitx2* induction in the right LPM or its wildtype expression in left injected specimens. Importantly, loss of *dand5* mRNA was already observed in pre-flow stages (Fig. 2F, Supplementary Fig. 2A), indicating independence of flow. Toxic effects by m-tpMOs were excluded because targeted sLRO cells depicted wt *nodal1* expression (Supplementary Fig. 2A). Thus, we identified a medially localized sub-region in the Bicc1RE, likely required for *dand5* mRNA stability. Intriguingly, left-sided d-tpMO injections did not alter flow-induced downregulation of *dand5* mRNA (Fig. 2G, H), although *pitx2* asymmetry was lost. Irrespective of which side was targeted, the frequency of stronger right-sided *dand5* signals compared with left sLRO (R>L) in post-flow stages did not differ between untreated controls and d-tpMO injected specimens (Fig. 2G, H). In pre-flow embryos (st. 16) no changes in *dand5* expression were observed either (Fig. 2I, Supplementary Fig. 2C). *nodal1* expression was also not altered, showing that d-tpMOs did not cause unspecific detrimental effects (Supplementary Fig. 2D). Our results suggest that the distal sub-region conveys flow-dependent repression specifically via *dand5* translation but does not impair mRNA decay.

### Bicc1 regulates *dand5* and *nodal1* expression at pre-flow stages

To connect our observed tpMO effects on the *dand5* Bicc1RE to Bicc1 function, we performed *bicc1* LoF experiments. *X. laevis* offers precise targeting of sLRO cells by microinjection of the left or right C2-lineage^26–28^, whereas avoiding the flow-generating cLRO (Supplementary Fig. 3A). This injection setup circumvents described defects in cilia polarization. To knockdown *bicc1*, a previously published TBMO^20^ was used as well as a designed splice-blocking MO (SBMO). In both cases, two MOs were used which specifically targeted the S- or L-allele, which are both expressed during embryogenesis and encode identical proteins^29^. Injecting either MO separately did not affect laterality (Supplementary Fig. 3B).

**Fig. 3:**
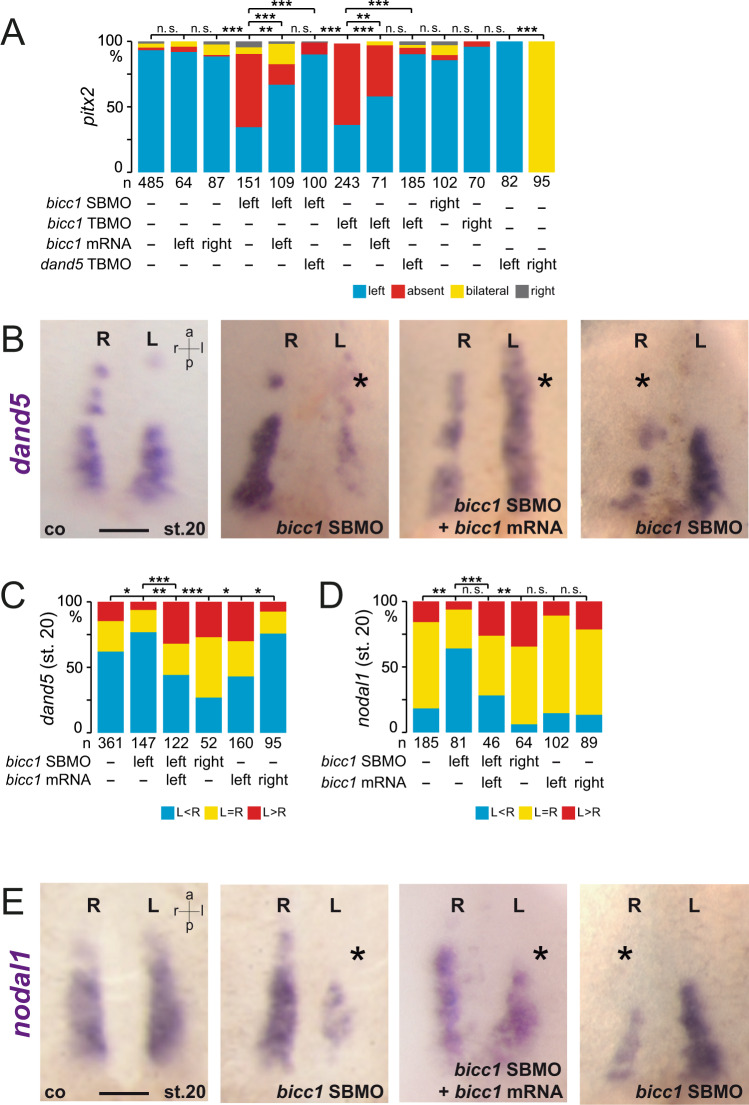
Bicc1-dependent *dand5* and *nodal1* expression in sLRO cells. **A** Absence of left LPM *pitx2* expression in *bicc1* morphants, unilaterally injected on the left, was rescued by parallel knockdown of *dand5*. Specificity of TBMO or SBMO was shown by co-injecting rescue mRNAs, i.e., mouse *bicc1* or *Xenopus bicc1*, respectively. Note *dand5* knockdown on the right efficiently induced *pitx2* expression, as published. **B** Loss of *dand5* mRNA at post-flow stages (st. 20) following left- and right-sided *bicc1* SBMO injections. Controls (co) showed wt expression. *dand5* expression was restored by co-injecting *bicc1* rescue mRNA. Note enhanced *dand5* staining in rescued specimens. **C** Quantification of *dand5* expression after knockdown of *bicc1*. The effect was observed in the left and right sLRO cells. **D**, **E** Downregulation of *nodal1* in *bicc1* morphants. **D** Quantification of results. **E** Wt specimens show bilateral *nodal1* mRNA. Left or right *bicc1* SBMO injections reduced *nodal1*, which was restored by adding rescue *bicc1* mRNA. MO pmol/embryo: *bicc1* SBMO (L and S, each 1); *bicc1* TBMO, (L and S, each 1); *dand5* TPMO (0.5). Asterisks in **B** and **E** mark the injected side. Numbers (n) in **A**, **C**, and **D** represent analyzed specimens from more than three independent experiments. Statistical analyses were done with one-sided Pearson’s chi-square test, which was adjusted for multiple comparisons by Bonferroni (**B**) or Bonferroni–Holm (**C**, **D**). n.s. not significant *p* > 0.05; * significant, *p* < 0.05; ** highly significant *p* < 0.01; ***, very highly significant *p* < 0.001. *p* values and listing of individual experiments can be found in the source data file. st., stage. Scale bars in **B** and **E** represent 100 µm. st. stage, a anterior, l left, r right, p posterior.

In morphants, in which the sLRO was targeted by co-injection of S- and L-MOs, the LRO morphology and cilia polarization in cLRO and sLRO cells was unaffected, demonstrating proper targeting (Supplementary Fig. 3C, C’, D). *pitx2* expression, however, was predominately absent in morphants injected unilaterally on the left side (Fig. 3A; Supplementary Fig. 2E). Right-sided *bicc1* LoF had no effect on *pitx2* expression. MO-specificity was demonstrated by co-injecting full-length *bicc1* mRNA that was not targeted by either MO (mouse *Bicc1*, *mbicc1*, in case of TBMO, and *Xenopus bicc1* in case of SBMO), which rescued *pitx2* expression in a significant proportion of specimens (Fig. 3A; Supplementary Fig. 3E). In addition, splicing of *bicc1* pre-mRNA was affected in SBMO-treated specimens, shown by RT-PCR (Supplementary Fig. 3F). Interestingly, GoF alone did not affect *pitx2* (Fig. 3A), which indicates that an excess of Bicc1 was not interfering with flow sensing or subsequent processes. Both *bicc1* MOs gave virtually identical results, fulfilling yet another criterion for the controlled use of MOs^30^. Importantly, parallel LoF of *bicc1* and *dand5* in left sLRO cells rescued *pitx2* expression (Fig. 3A), which fits a scenario where Bicc1 acts downstream of flow and upstream of flow-mediated *dand5* repression.

We, therefore, analyzed the expression of the flow target *dand5* in post-flow *bicc1* morphants. We observed a strong downregulation of *dand5* mRNA (Fig. 3B, C), instead of the expected loss of *dand5* repression and blocked mRNA decay. This effect was not restricted to left sLRO cells as right-sided MO injections equally led to *dand5* mRNA reduction (Fig. 3C). In pre-flow stages, *dand5* was also downregulated (Supplementary Fig. 3G). Importantly, *dand5* expression was restored in *bicc1* morphants by co-injecting *bicc1* rescue mRNAs, demonstrating specificity (Fig. 3B, C). Injecting *bicc1* mRNA alone or with MOs boosted *dand5* mRNA expression in left or right sLRO cells (Fig. 3B, C), which hinted towards an enhanced *dand5* mRNA stability or expression. Overall, the *bicc1* MO phenotype on *dand5* mRNA clearly resembled the results obtained when the *dand5* 3′-UTR was targeted by the m-tpMO (Fig. 2D, E; Supplementary Fig. 2A). However, loss of *dand5* mRNA by *bicc1* LoF was not congruent with observed effects on *pitx2*, i.e., no ectopic right-sided induction but the loss of left-sided *pitx2*, suggesting that additional factors were affected.

To explore this further, we monitored *myo1d* and confirmed that the positioning and the somitic identity of sLRO cells were unaltered in *bicc1* morphants, (Supplementary Fig. 3H), excluding a general failure in specification or morphogenesis. We then analyzed *nodal1* in sLRO cells. Like *dand5*, *nodal1* expression was substantially reduced in *bicc1* morphants at pre- and post-flow stages (Fig. 3D, E; Supplementary Fig. 3I). The effect was specific because *nodal1* expression was rescued by co-injections of *bicc1* mRNAs (Fig. 3D, E), suggesting that this effect contributed to the observed LR defects, i.e., loss of *pitx2* expression. Bicc1 regulation of *nodal* mRNA has not been reported previously. Taken together, Bicc1 controls the expression of both key effectors of symmetry breakage independent of leftward flow.

### Bicc1 ensures *gdf3* mRNA translation and thereby *nodal1* expression

**Fig. 4:**
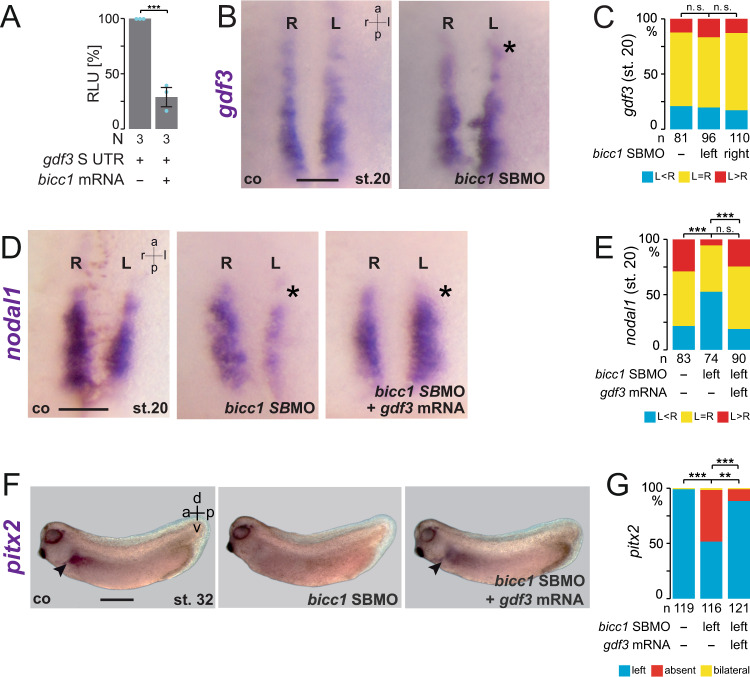
Bicc1 indirectly regulates nodal1 expression via Gdf3 signaling. **A** Animal cap assay using a *luciferase* reporter mRNA which contained *gdf3* 3′-UTR sequences. Translation of *gdf3* reporter was efficiently blocked by co-injecting *bicc1* mRNA. N represents the number of independent experiments. A pool of 10 animal caps was analyzed per experiment and treatment. The result from reporter mRNA alone served as a reference and was set to 100% RLU. Relative values of single experiments are depicted as blue dots. Data of three experiments are presented as mean value (bar) ±standard deviation (error bar, SD). Statistical analyses were done with a one-sided Student’s *t* test for two independent means using the values of three individual experiments. **B** *gdf3* mRNA was not affected by *bicc1* LoF. **C** Quantification of *gdf3* expression in *bicc1* morphants at the LRO margin. **D** *gdf3* GoF rescues *nodal1* expression in *bicc1* morphants. Representative *nodal1* staining in left sLRO cells is shown for control (co), *bicc1* morphant, and rescued specimens. **E** Quantification of the *bicc1* MO rescue of *nodal1* expression by *gdf3*. **F**, **G** Left-asymmetric *pitx2* expression (arrowhead) is restored in *bicc1* morphants by co-injecting *gdf3* mRNA. MO pmol/embryo: *bicc1* SBMO (L and S, each 1). Asterisks in **B** and **D** mark injected side. Numbers (n) in **C**, **E**, and **G** represent analyzed specimens from more than independent experiments. Statistical analyses were done with one-sided Pearson’s chi-square test, which was adjusted for multiple comparisons by Bonferroni–Holm (**C**, **E**) or Bonferroni (**G**). n.s. not significant, *p* > 0.05; ** highly significant *p* < 0.01; ***, very highly significant *p* < 0.001. *p*-values, mean values, SD and listing of individual experiments can be found in the source data file. Scale bars in **B** and **D** represent 100 µm and in **F** 1 mm. RLU relative luciferase units, st. stage, a anterior, l left, r right, p posterior, d dorsal, v ventral.

Previous reports suggested that *nodal1* is regulated by Gdf3 signaling in sLRO cells^31^ and that *gdf3* is post-transcriptionally regulated^32^. To test whether *gdf3* might be regulated by Bicc1, a luciferase reporter containing the *gdf3* 3′-UTR (361 bp S alloallele) was analyzed in the AC assay. AC cells are devoid of endogenous *gdf3* expression. Translation of *gdf3* reporter mRNA was significantly repressed by Bicc1 (Fig. 4A). Unlike *dand5* and *nodal1*, *gdf3* mRNA in sLRO cells was not altered in *bicc1* SBMOs injected specimens (Fig. 4B, C). *nodal1* mRNA was diminished in *gdf3* morphants leading to impaired *pitx2* asymmetry (Supplementary Fig. 4), suggesting that Bicc1 could act on *gdf3* translation in vivo. If loss of *nodal1* mRNA in *bicc1* morphants was owing to impaired Gdf3 signaling, *nodal1* expression should be restored by *gdf3* GoF. Co-injecting *bicc1* SBMOs and *gdf3* mRNA in the left sLRO lineage indeed rescued *nodal1* expression as well as *pitx2* asymmetry (Fig. 4 D, E and F, G respectively), demonstrating that Bicc1 enabled Gdf3-dependent *nodal1* expression. Thus, in pre-flow stages, Bicc1 seems to be required for securing the interplay of secreted key factors (Nodal1/Gdf3/Dand5) until positional information is provided by leftward flow which represses *dand5*.

### Bicc1 acts in a context-dependent manner with sub-regions of the Bicc1RE

**Fig. 5:**
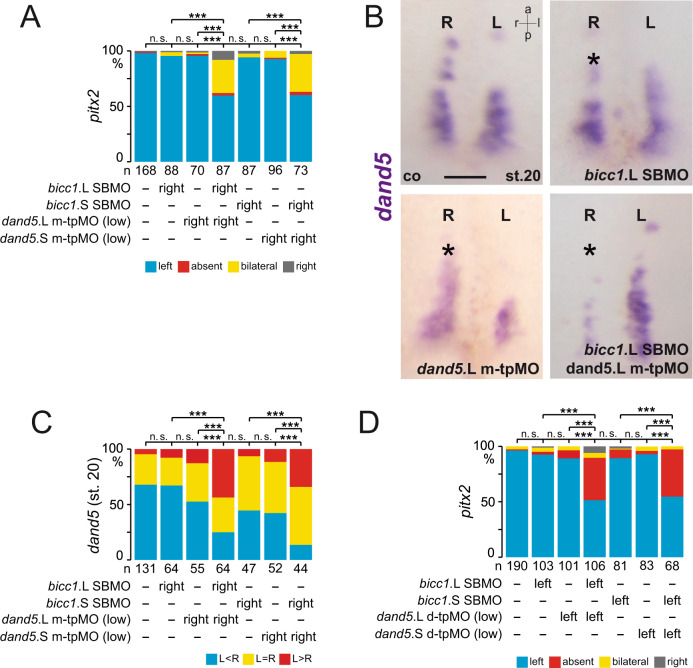
Bicc1 regulates *dand5* mRNA through distinct regions of the Bicc1RE. **A** Quantification of right-sided *pitx2* induction by co-injecting a low, ineffective m-tpMO dosage with single allele-specific *bicc1* SBMO. Controls (co), m-tpMO (S or L, low), or allele (S or L) specific *bicc1* SBMO alone showed wt *pitx2* asymmetry. **B** Co-injecting m-tpMO (low) with *bicc1* TPMO (L or S) impacted *dand5* mRNA stability. Treatment with low concentrations of m-tpMO, single allele-specific *bicc1* TPMO, and uninjected co showed wt *dand5* expression at post-flow stages. **C** Quantification of *dand5* expression. **D** Quantification of *pitx2* asymmetry. Only in combination both suboptimal dosages of d-tpMO (low) or single allele-specific *bicc1* SBMO (S or L) prevented left *pitx2* expression. Wt expression was found in controls (co) and in embryos that were left-sided injected with one MO alone. MO pmol/embryo: *bicc1* SBMO (L or S, 1); m-tpMO low (L or S, 0.4); d-tpMO low (L or S, 0.5). Asterisks in **B** mark injected side. Numbers (n) in **A**, **C**, and **D** represent analyzed specimens from more than independent experiments. Statistical analyses were done with one-sided Pearson’s chi-square test, which was adjusted for multiple comparisons by Bonferroni (**A**, **D**) or Bonferroni–Holm (**C**). n.s., not significant *p* > 0.05; ***, very highly significant *p* < 0.001. *p* values and listing of individual experiments can be found in the source data file. Scale bar in **B** represents 100 µm. st. stage, a anterior, l left, r right, p posterior.

To provide further evidence that Bicc1 regulation of *dand5* is relayed through the Bicc1RE, we tested for functional cooperation of *bicc1* SBMOs with tpMOs. Consequently, we used single L- or S-specific *bicc1* SBMOs which, separately, did not impact on *pitx2*, *dand5,* and *nodal1* expression, in combination with suboptimal dosages of tpMOs (Fig. 5A–D; Supplementary Fig. 3B, 4A–C). Right-sided injection of low dose m-tpMO (S or L) together with a single *bicc1* SBMO (S or L) resulted in ectopic right-sided LPM induction of *pitx2* (Fig. 5A; Supplementary Fig. 5A), mimicking treatments with high dose m-tpMO (Fig. 2A, B). Accordingly, double morphants showed reduced *dand5* expression on the injected side (Fig. 5B, C). These results suggested that in early neurulae *dand5* mRNA stability depends on (a) Bicc1 and (b) the accessibility of the medial sub-region of the Bicc1RE.

Next, we performed alike experiments with d-tpMOs, to prove the involvement of Bicc1 in post-flow *dand5* regulation. Indeed, left-sided injections of low doses of d-tpMO together with allele-specific *bicc1* SBMO prevented *pitx2* induction in the left LPM (Fig. 5D; Supplementary Fig. 5B). Importantly, *nodal1* expression in sLRO cells was normal (Supplementary Fig. 5C), unlike in *bicc1* morphants (Fig. 3D, E). We, therefore, concluded that flow-induced *dand5* translational repression required Bicc1 activity, which merged on the distal sub-region of the Bicc1RE. Taken together, the cooperation experiments of *bicc1* SBMOs and tpMOs underscore a dual role of Bicc1 on flow independent *dand5* mRNA stability or its flow-induced translational repression. Intriguingly, both Bicc1 functions converge on a small 3′-UTR sequence, the Bicc1RE.

### Bicc1 and Dicer interact in post-transcriptional *dand5* regulation

**Fig. 6:**
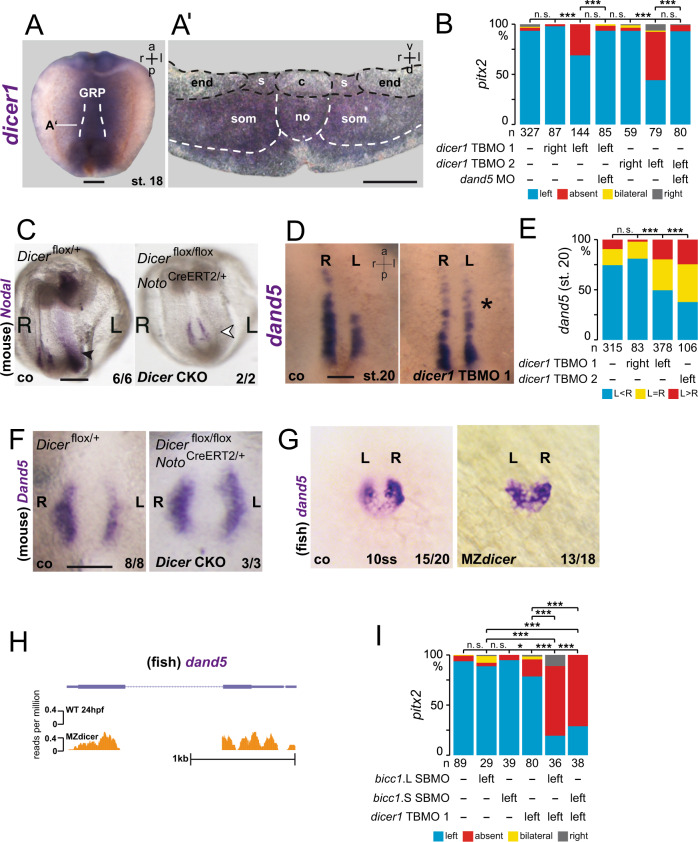
Dicer interacts with Bicc1 in *dand5* repression. **A** Expression of *dicer1* in sensory (s) LRO cells ( N = 3; n = 30) of the frog (GRP; gastrocoel roof plate). Whole-mount in situ hybridizations of stage 18 dorsal explant with a *dicer1*-specific antisense RNA probe. (A’) The transverse histological section (indicated in **A**) reveals mRNA expression in sLRO cells, somites (som), and deep cells of the notochord (no), but absence of signals from central (c) flow-generating LRO and lateral endodermal cells (end). **B** Quantification of MO-mediated inhibition of *dicer*. Note knockdown in left, but not right sLRO cells prevented *pitx2* asymmetry in the left LPM, which was rescued by co-injecting *dand5* MO. **C** mRNA expression of *nodal* in control (*Dicer*^flox/+^) and *dicer* conditional knockout (*Dicer*^flox/flox^ *Noto*^CreERT2/+^) mouse embryos at E8.0. Note that *Nodal* asymmetry in the left LPM (arrowhead) was lost in mutants. **D** Absence of flow-induced *dand5* mRNA decay at the left LRO margin in post-flow *dicer1* morphants (st. 20). Representative dorsal explants of wt (left) and *dicer1* morphant (right) specimens hybridized with a *dand5* antisense RNA probe. **E** Quantification of *dand5* results. **F** Flow-induced *Dand5* mRNA downregulation in left crown cells of the murine node was lost in *Dicer* conditional knockout (*Dicer*^flox/flox^ *Noto*^CreERT2/+^) mouse embryos at E7.5. **G** Lack of *dand5* repression in 10 somite stage (ss) MZdicer mutant zebrafish embryos. **H** Absence of *dand5* mRNA by RNAseq reads in 24hpf wt zebrafish embryos, but maintenance in MZ*dicer* mutants. **I** *bicc1* and *dicer1* interact in LR asymmetry. Wt *pitx2* expression upon isolated left-sided injections of allele-specific *bicc1* SBMOs and moderate effects upon *dicer1* TBMO1 injection. Asymmetric *pitx2* was significantly inhibited by co-injecting *dicer1* and *bicc1* MOs. MO pmol/embryo: *dicer1* TBMO1 (1.5); *dicer1* TBMO2 (1); *bicc1* TBMO (L or S, each 1); *bicc1* SBMO (L or S, each 1). Asterisks in **D** mark injected side. Numbers (n) in **B**, **E**, and **I** represent analyzed specimens from three independent experiments. Statistical analyses were done with one-sided Pearson’s chi-square test, which was adjusted for multiple comparisons by Bonferroni (**B**, **I**) or Bonferroni–Holm (**E**). n.s. not significant *p* > 0.05; * significant, *p* < 0.05; ***, very highly significant *p* < 0.001. *p* values and listing of individual experiments can be found in the source data file. Scale bars in **A**, **A**’, **C**, **F**, and **D** represent 100 µm. st. stage, a anterior, d dorsal, l left, r right, v ventral, p posterior.

Several reports have shown Bicc1 regulation of miRs, small RNAs that bind specific 3′-UTRs and thereby tag the mRNA for translational repression and decay. The RNase III enzyme Dicer processes precursor miRs in the cytoplasm and, together with Ago2, assembles the RNA-induced silencing complex RISC^33^. In the kidney, Bicc1 acted downstream of Dicer1 to transfer target mRNAs unto Ago2, which cleaves or blocks their translation in a miR-dependent manner^22^. To begin exploring the possible role of miRs in *dand5* regulation, we analyzed the expression of *dicer1*. *dicer1* mRNA was expressed in somites and notochord at flow-stage (st. 18; Fig. 6A). *dicer1* mRNA was found specifically in sLRO cells, excluding the cLRO cells in-between and the lateral endodermal cells flanking the LRO (Fig. 6A, A’). Two MOs that targeted translation (TBMO1^34^; TBMO2) through conserved sequences of both S- and L-alleles were used to knockdown *dicer*. Targeting the left side of the LRO (C2-lineage) inhibited *pitx2* expression in the left LPM (Fig. 6B). Wildtype phenotypes upon right-sided MO injections argue against MO toxicity and off-target effects (Fig. 6B). A parallel knockdown of *dand5* on the left rescued wt *pitx2* expression (Fig. 6B; S6A), further supporting MO-specificity. In addition, western blot analysis confirmed the efficacy of the designed *dicer1* TPMO2 (Supplementary Fig. 6B). In mouse embryos, *Dicer* was also required for Nodal cascade asymmetry. Induced conditional deletion of *Dicer* from the mouse LRO prevented the expression of *Nodal* mRNA in the left LPM (Fig. 6C).

Analyzing earlier stages of laterality determination, left-sided downregulation of *Dand5* mRNA levels at post-flow stages was compromised in mouse *Dicer* mutants and *Xenopus dicer1* morphants (Fig. 6D, E, F). This finding was conserved in zebrafish. In wt 10 somite stage (ss) embryos, *dand5* was repressed on the left side of Kupffer’s vesicle (KV), whereas no repression was observed in maternal zygotic *dicer* mutants (MZ*dicer*; Fig. 6G). At the wt KV *dand5* mRNA fades away at 14 ss and is absent at 18 ss (Supplementary Fig. 6C; source data file). Although MZ*dicer* mutants showed some developmental delay, *dand5* expression was retained as late as 24hpf, which was monitored by ISH (Figure S6C) and RNAseq (Fig. 6H; Supplementary Fig. 6D, E). Loss of *dand5* repression upon *dicer* LoF could be caused by the absence of flow or represent a specific function on *dand5* regulation. Previous reports have shown that miRs control motile ciliogenesis^35,36^. In agreement with this, ciliation of multiciliated cells in the *Xenopus* epidermis was impaired in *dicer1* morphants (Supplementary Fig. 6H). When *dicer1* MOs were targeted to flow-generating LRO cells (C1-lineage), ciliation was unaltered in morphants (Supplementary Fig. 6F, F’, G), demonstrating that *dicer* acted downstream of flow and upstream of *dand5* repression, like *bicc1*. Next, we investigated whether *dicer1* and *bicc1* acted in the same pathway in flow-sensing cells. When injecting *bicc1* SBMOs (targeting S- or L-alleles) separately, wt *pitx2* induction in the left LPM was observed (Fig. 6I; Supplementary Fig. 3B). Co-injection of either *bicc1*.S- or L-SBMO and *dicer1* MO blocked *pitx2* expression in ~70% of cases (Fig. 6I), demonstrating that *bicc1* and *dicer1* synergize to mediate *dand5* repression.

**Fig. 7:**
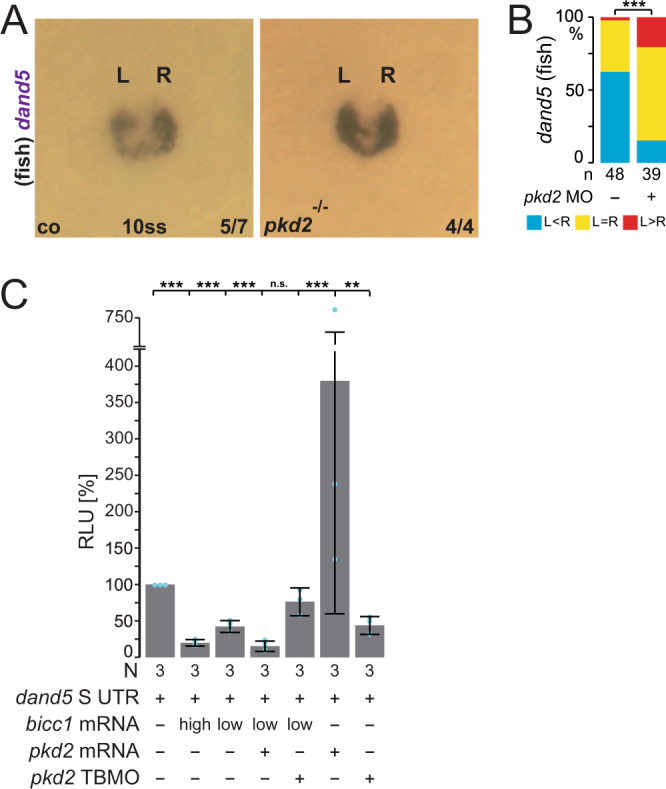
Pkd2 function modifies Bicc1-mediated translational repression of *dand5*. **A** Absence of *dand5* repression in maternal zygotic (MZ) *pkd2* mutant zebrafish at 10 somite stage (ss). **B** Quantification of *dand5* asymmetry in controls (co) and *pkd2* morphant (1–4 ng) zebrafish. Asymmetry was determined by picture analysis using ImageJ. Number (n) represents the number of analyzed specimens. Statistical analyses were done with one-sided Pearson’s chi-square test. **C** Animal cap luciferase reporter assay of full-length *dand5.S* 3′-UTR (cf. Figure 1A). The reporter construct was injected as mRNA either alone or in combination with high or low dose *bicc1* mRNA, *pkd2* mRNA or *pkd2* TBMO. Gradual repression upon co-injection of high or low concentrations of *bicc1* mRNA was observed. Administering only *pkd2* mRNA or *pkd2* TBMO (1 pmol) efficiently blocked or boosted luciferase expression, respectively. The data further indicate that in AC cells endogenous *dand5* mRNA is post-transcriptionally regulated in a Pkd2-dependent manner. In the presence of a lower amount of *bicc1* mRNA high-level, strong repression was achieved when *pkd2* mRNA was co-injected, or further diminished upon knockdown of *pkd2* using TBMO. N represents the number of independent experiments. A pool of 10 animal caps was analyzed per experiment and treatment. The results from reporter mRNA alone served as reference and were set to 100% RLU. Relative values of single experiments are depicted as blue dots. Data of three experiments are presented as mean value (bar) ±standard deviation (error bar, SD). Statistical analyses were done with a one-sided Student’s *t* test for two independent means (Bonferroni corrected) using the values of three individual experiments. *p* values, values for individual experiments, mean values, and standard deviations are found in the source data file. n.s. not significant, *p* < 0.05; ** highly significant *p* < 0.01; ***, very highly significant *p* < 0.001, RLU relative luciferase units, *Luc* *luciferase*.

Finally, we wondered whether *pkd2*, one of two published active components in the flow sensor^37,38^, acted in the same pathway. Our recent demonstration of an earlier (likely maternal) Pkd2 function in the specification and morphogenesis of the LRO prevented us from investigating this question in the context of LR axis formation in the embryo itself^28^. In zebrafish, however, zygotic *pkd2* mutants and morphants display randomization of *nodal* (*southpaw*), *lefty* and *pitx2*, but are reported to have normal KV ciliation and morphology^39,40^, suggesting a role for Pkd2 in flow sensing. In agreement with this, *dand5* mRNA repression was not observed in *pkd2* mutant and morphant zebrafish embryos (Fig. 7A, B), likely being causative for misregulation of the Nodal cascade in these backgrounds^39^. To test a potential interplay between *pkd2* and *bicc1* in the process of *dand5* repression, we returned to the *Xenopus* AC assay (Fig. 1A). In order to record additive effects of *pkd2*, we attenuated the Bicc1-mediated repression of the *dand5* reporter by lowering the concentration of co-injected *bicc1* mRNA, such that reporter activity was only repressed to ~40% of wt-level (Fig. 7C). Upon co-injection of full-length *pkd2* mRNA, reporter activity was further repressed to under 20% (Fig. 7C). *pkd2* mRNA alone, however, increased the reporter mRNA’s expressivity fourfold. Because *pkd2* is maternally expressed in animal tissue, like *dand5*, we tested this interaction further by co-injecting *pkd2* MO, the specificity of which we showed previously^17,41^. Loss of *pkd2* partially rescued *bicc1*-mediated repression of the *dand5* reporter (Fig. 7C), which again is contrasted in a *bicc1* free set up, where *pkd2* is required for efficient translation. Therefore, these experiments underscore a scenario in which an upstream ion-channel Pkd2 is able to modulate Bicc1 function during post-transcriptional regulation of *dand5*.

In summary, data presented here demonstrate that the proximal *dand5* 3′-UTR contains regulatory sequences, which allow the RNA-binding protein Bicc1 and the miR-processing enzyme Dicer to execute flow-dependent *dand5* repression. Thereby this Bicc1RE likely reflects the downstream target of calcium released by Pkd2 in sensing of leftward flow at the left LRO margin.

## Discussion

**Fig. 8:**
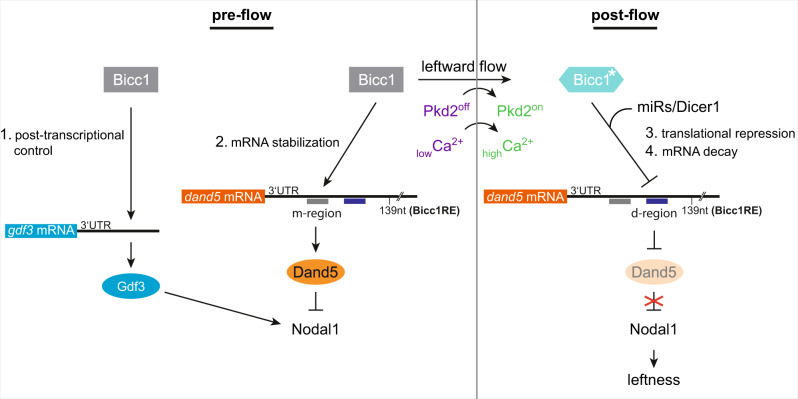
Two modes of Bicc1-dependent post-transcriptional regulation of *gdf3* and *dand5* in flow sensor cells at the *Xenopus* left-right organizer. In the early neurula pre-flow stages, Bicc1 has two functions. Bicc1 assures *gdf3* mRNA translation and thereby indirectly ensures *nodal1* transcription by Gdf3 signaling. Simultaneously Bicc1 mediates *dand5* mRNA stability via the medial (m) sub-region of the Bicc1RE. Thus, Dand5 protein levels are sustained on both sides, keeping Nodal in tight repression. Leftward flow activates the Pkd2 channel in left flow sensor cells, resulting in an asymmetric calcium signal. In post-flow stages, a calcium-dependent mechanism activates/modifies Bicc1 to become a repressor of *dand5* translation, which is relayed by the distal (d) sub-region of the Bicc1RE. Subsequently, *dand5* mRNA gets degraded in a Dicer1 (miR) dependent manner. Attenuated Dand5 expression lifts repression of Nodal and defines leftness by induction of the LPM Nodal signaling cascade. For details, see text.

We have identified a minimal 139 nt sequence in the *dand5* 3′-UTR, which was sufficient to mediate Bicc1-dependent post-transcriptional regulation. This Bicc1RE contains two sub-regions, medial and distal, which represent two distinct regulatory entities in a pre- and post-flow setting. In pre-flow stages, free access to the medial sub-region and a sufficient amount of Bicc1 protein was required to maintain *dand5* mRNA expression. In this  context, Bicc1 might protect *dand5* against premature mRNA decay and ensures Dand5 protein synthesis. However, observed LR defects in *bicc1* morphants were to a great extent caused by loss of *nodal1* expression, which so far has not been reported in other organisms. We identified an additional potential Bicc1 target, the Tgfβ ligand *gdf3*. This was evidenced by the efficient repression of the *gdf3* 3′-UTR reporter mRNA by Bicc1 (Fig. 4A). However, *gdf3* mRNA expression, unlike *dand5* and *nodal1*, was not impaired by *bicc1* LoF indicating that in this case, Bicc1 acted on translation only. Gdf3 is required for efficient Nodal diffusion and therefore LR patterning^16,31,42,43^. In *Xenopus* sLRO cells an additional *gdf3* function was reported, suggesting that Gdf3 signaling is upstream of *nodal1* transcription^32^, which we validated in this study (Supplementary Fig. S4A, B). This finding was underscored by our observation that *gdf3* overexpression restored *nodal1* expression in *bicc1* morphants. Currently, there is no evidence of such a mechanism in other vertebrates and it likely reflects a frog-specific feature. We propose a pre-flow situation in which Bicc1 safeguards the expression of *dand5* and *gdf3* in a post-transcriptional manner. Thus, it indirectly influences *nodal1* transcription via Gdf3 signal transduction (Fig. 8). In the embryonic kidney of *Xenopus*, a comparable protective Bicc1 function was shown for *pkd2*^17^. Such a scenario should be relevant to ensure an at least equimolar equilibrium of the inhibitor Dand5 and its targets in sLRO cells. In addition, Bicc1 control of *gdf3* limits or prevents ectopic Nodal signaling and premature Nodal/Gdf3 diffusion until flow sensing.

Early Bicc1 functions impeded the analysis of flow-dependent *dand5* mRNA regulation, but we were able to show several lines of evidence that Bicc1 serves as the critical mediator of flow sensing and primarily blocks *dand5* translation. (1) Rescue of *pitx2* asymmetry in *bicc1* morphants by *dand5* knockdown demonstrated that even when *dand5* and *nodal1* were strongly reduced, *dand5* was not repressed in absence of Bicc1. (2) Using our d-tpMOs, we separated *dand5* mRNA decay from translation inhibition: left-sided, flow-dependent *dand5* mRNA reduction was still observed in d-tpMO morphants (Fig. 2G, H), whereas left nodal cascade induction was inhibited (Fig. 2A, C). (3) Bicc1 dosage and availability of distal sequences of the Bicc1RE cooperated in flow-dependent *dand5* repression and left *pitx2* LPM induction, without any effects on dand5 mRNA stability (Fig. 5D; Supplementary Fig. 5D). (4) The accompanying manuscript by Minegishi et al.^44^ demonstrates Bicc1 binding to the mouse *Dand5* 3′-UTR and identified specific binding motifs. Intriguingly, we found alike sequences in the Bicc1RE, which apparently were located within dS-tpMO or next to dL-tpMO target sequences that specifically impair translational repression in *Xenopus* (Figure S1B). In addition, deleting the distal 36 nucleotides of the Bicc1RE, which contains the site, renders the *dand5* reporter mRNA insensitive to Bicc1 (Fig. 1D). Based on the conserved nature of *dand5* as the flow target, the finding in mouse should also apply to *Xenopus*. Potential sites were also found in the *gdf3* 3′-UTR, underscoring their relevance. (5) Both Bicc1 and Dicer are well known for post-transcriptional regulation and they functionally interacted in flow-induced *dand5* repression (Fig. 6I). (6) The flow sensor *pkd2* was able to modulate Bicc1 properties on *dand5* translational inhibition, suggesting that calcium could serve as the switch from a safeguarding pre-flow to a modified inhibitory post-flow Bicc1 function.

Our work, together with complementing analyses in the mouse (cf.^44^), constitutes a conceptual advance in our understanding of symmetry breaking, namely the flow-dependent activation of the RNA-binding protein Bicc1 to repress *dand5* translation on the left LRO margin in a Dicer-dependent manner. Based on our analyses, we suggest a model schematically depicted in Fig. 8. In the pre-flow scenario, Bicc1 protects *dand5* mRNA in a bilateral symmetric manner, which is relayed by the medial sub-region of the Bicc1RE. Thereby, Dand5 synthesis and Nodal inhibition are secured. During flow, left-sided Pkd2 channel activation results in a cytoplasmic Ca^2+^ signal, which has been described in mouse and zebrafish^10,11,38,45,46^. It represents the intracellular second messenger of the initially extracellular flow signal. In zebrafish, transient activation of CaMK-II downstream of asymmetric Ca^2+^ is required in the LRO for asymmetric Nodal cascade induction and correct development of organ situs^47^. We hypothesize that Bicc1 gets functionally modified (Bicc1*) by Pkd2 and potentially Ca^2+^, which alters Bicc1 properties from initial *dand5* mRNA stabilization to translation inhibition, followed by mRNA decay (Fig. 8). How this molecular switch is achieved remains unclear. In *Drosophila,* Bicc1 phosphorylation has been reported and therefore Ca^2+^-dependent phosphorylation might lead to functional changes^48^. Then again, under certain conditions, Bicc1 is thought to form polymeric complexes. Recently it was speculated that monomeric or polymeric Bicc1 aggregates may act differentially on post-transcriptional regulation. It was proposed that in left sLRO cells Bicc1 polymerization might be induced in a *pkd2*/Ca^2+^-dependent manner that blocks *dand5* translation, whereas on the right side only low molecular Bicc1 complexes are present, allowing Dand5 synthesis^49^. So far, we do not have any evidence of how Bicc1 is modified by the leftward flow. However, our analysis demonstrated that the functional switch is accompanied by a differential requirement of relevant sub-regions in the 3′-UTR of *dand5*. This finding may be very useful in the future to map crucial Bicc1 domains and sequences for *dand5* inhibition.

In evolutionary terms, the *pkd2*/*bicc1*/*dicer* module is functionally conserved from zebrafish to mammals. In mouse and *Xenopus*, a proximal element of the *dand5* 3′-UTR is required and sufficient for flow-mediated mRNA decay and translational inhibition, respectively, which is dependent on Bicc1 and Dicer (cf.^44^). Whether or not miRs are involved in Dicer1-mediated *dand5* repression remains open. The analysis of the proximal regions of various vertebrate *dand5* 3′-UTR sequences, which show the highest degree of conservation, using different miR-target prediction tools, detects only a few potential miR-binding sites, with low probabilities in all cases. However, miR-133 may be relevant, because members of this family are specific for muscle development and expressed in somites and sLRO cells have somitic fate^8,50–52^. A conserved target site was detected in the Bicc1RE of *X. laevis* S- and L-alleles and the human proximal *dand5* 3′-UTR (Figure S1B; Supplementary Fig. 7A). It remains to be seen whether one of the four family members in *Xenopus* is involved in Bicc1-mediated *dand5* mRNA stability and post-flow repression. Interestingly, Bicc1 regulates its own expression in a post-transcriptional manner^53^. A highly conserved miR-133-binding site in 3′-UTRs of vertebrate *bicc1* genes (Supplementary Fig. 7A, B, C) may suggest that a Bicc1/miR-133 module has been adapted to the regulation of *dand5* in somitic/sLRO cells. Alternatively, Dicer may act miR-independently through one of its described non-canonical mechanisms^54^.

In conclusion, our work identified Bicc1 and Dicer as two factors downstream of leftward flow sensing. The exact nature of Bicc1’s modifications and interactions with the *dand5* Bicc1RE in a pre-flow and post-flow setting remains to be solved.

## Methods

### Image processing

Imagej (1.48i), Acrobat Illustrator (cs6), and Acrobat Photoshop (cs6) were used for image processing.

### Plasmid construction

The m*bicc1*-CS2+ construct was a gift from Oliver Wessely (Cleveland, OH, United States).

For in vitro synthesis of mRNA using the Ambion sp6 message kit, the plasmids were linearized with NotI.

Firefly luciferase reporter mRNAs that contained the *gdf3* mRNA 3′-UTR (GenBank: BC073508.1) or the 3′-UTR of the *dand5*.L mRNA or the *dand5*.S mRNA the Ambion T7 message kit were used and the plasmids were linearized with BamH1.

Supplemental Table 2 lists all primers with sequences used in the context of this work.

### RT-PCR

RT-PCR was conducted using either the L or S isoform-specific primer for intron 2 or intron 1, respectively, and an isoform-specific reverse primer in exon 5 with 38 cycles. The listing of individual primers used in this work can be found in Supplemental Table 2.

### Morpholinos

Supplemental Table 1 lists all MOs used with references to previous validations or proof of specificity in the context of this work.

### *Xenopus* frogs and embryos

Animals were handled in accordance with German regulations (Tierschutzgesetz) and approved by the Regional Council Stuttgart (A379/12 Zo, ‘Molekulare Embryologie’, V340/17 ZO and V349/18 ZO, ‘*Xenopus* Embryonen in der Forschung’).

*Xenopus* embryos obtained by in vitro fertilization were maintained in 0.1× modified Barth medium^55^ and staged according to ref. ^56^. During injections, embryos were kept in 1× modified Barth medium with 2% Ficoll. To specifically target the sensory cells of the GRP for all experiments except for the luciferase assay, we injected them into the dorsal marginal side (left or right; C2-lineage). For luciferase assays, embryos were injected twice into the animal blastomeres at the four-cell stage with a luciferase *dand5* 3′-UTR construct, alone or together with a *bicc1* construct. Animal cap tissue was dissected at stage 10 (cf. Figure 1A for a schematic depiction of the procedure). Following injections, all embryos were transferred to 0.1 modified Barth medium.

### Zebrafish

Established husbandry protocols were adhered to, and experimental protocols were conducted, in accordance with the Princeton University Institutional Animal Care and Use Committee (IACUC) guidelines. Zebrafish strains utilized include pkd2/cup^tc321^ ^39^ and dicer1^hu715^ ^57^. The pkd2 AUG MO is described in ref. ^39^. Embryos were staged according to ref. ^58^. Embryos were raised at 28 °C and processed for injections. For all knockdowns, a morpholino mixture of ~1.8 nl was injected into the yolk of one-cell stage embryos. All morpholino mixtures contained Danieau’s Buffer and 0.5 mg/ml phenol red.

Embryos were fixed at the 10 ss stage in 4% paraformaldehyde (PFA) overnight at 4 °C. These embryos were washed with PBST (1× PBS containing 0.1% Tween 20), dechorionated, transitioned to 100% methanol, and stored at −20 °C for at least 1 day. The transition to methanol was done by performing 5-minute washes in 75% 1× PBST:25% methanol, 50% 1× PBST:50% methanol, 25% 1× PBST:75% methanol, and 100% methanol. The embryos were then transitioned into 1× PBST by performing 5 min washes in 25% 1× PBST:75% methanol, 50% 1× PBST:50% methanol, and 75% 1× PBST:25% methanol. Embryos were then washed four times in 1× PBST with 5 min per wash. Somite stage embryos were incubated for 1 min in 1× PBST containing 0.01 mg/ml Proteinase K (Sigma Aldrich, P2308) followed by a 20 min incubation in 1× PBST containing 4% PFA. These embryos were then washed five more times in 1× PBST with 5 min per wash. Blastula and gastrula stage embryos did not undergo this Proteinase K treatment, extra fixation with 4% PFA, or the extra five washes with 1× PBST. Embryos were incubated in HYB (50% formamide, 5× SSC, 500 µg/ml torula yeast RNA, 50 µg/ml heparin 0.1% Tween 20, and 9 mM Citric Acid (pH 6.0)) for 2 h at 68 °C. Embryos were then incubated overnight in HYB containing an ISH probe at 68 °C. The next day, embryos were washed at 68 °C in HYB, 75% HYB: 25% 1× SSC, 50% HYB: 50% 1× SSC, 25% HYB: 75% 1× SSC, and 1× SSC for 10 min each wash. Embryos were then washed twice in 0.1× SSC for 30 min each wash. The remaining washes were performed at room temperature. Embryos were washed in 75% 0.1× SSC: 25% 1× PBST, 50% 0.1× SSC: 50% 1× PBST, 25% 0.1× SSC: 75% 1× PBST, and 1× PBST for 5 min each wash. Next, embryos were incubated on a rocker for 2 h in 1× PBST containing 2 mg/ml bovine serum albumin (BSA) and 2% normal sheep serum (NSS). Embryos were then incubated overnight on a rocker in 1× PBST containing 2 mg/ml BSA, 2% NSS, and 1:3500 of Anti-Digoxigenin-AP (Roche, 11093274910). The next day, the embryos were washed quickly in 1× PBST followed by six additional 15 min 1× PBST washes on a rocker. Embryos were then washed three times in NTMT (0.1 M Tris-Cl ph 9.5, 0.1 M NaCl, 0.05 M MgCl_2_, 0.1% Tween 20) and stained with 5 µl of NBT (Roche, 11383213001) and 3.75 µl BCIP (Roche, 11383221001) per 1 ml of NTMT. Staining was stopped by washing the embryos three times with NTMT, a 5 min wash with 1× PBST, and a 4 °C overnight incubation in 1× PBST containing 4% PFA. The embryos were then transitioned to methanol using the same four-step PBST:methanol washes listed above. Embryos were stored at −20 °C or cleared in 2:1 Benzyl Benzoate:Benzyl Alcohol prior to imaging. Canada Balsam containing 10% methyl salicylate was used to mount cleared embryos on a slide. RNA ISH staining was visualized using a Leica DMRA2 microscope and images were acquired using a Leica DFC450 C camera. The following probes were used for the ISHs: *dand5*.

### ImageJ RNA ISH image analysis of Zebrafish embryos

Pictures taken of the U-shaped *dand5* domain were cropped in Adobe Photoshop into equal-sized regions of interest corresponding to the left and right sides of the domain. The center of the domain was used as the midline for generating the left and right domains, and the entire staining area was included in the subsequent quantification analysis. “Subtract Background” in ImageJ 1.48i was used to remove unwanted background signals and images were inverted such that a darker stain, relating to more RNA presence, would yield a higher intensity. A ratio was obtained by dividing the right intensity by the left intensity. In this analysis, a right-biased *dand5* domain would have a ratio of 1.1 or higher, a left-biased *dand5* domain would have a ratio of 0.9 or lower, and an equal *dand5* domain would have a ratio between 0.9 and 1.1. At each stage and condition noted, the indicated number of embryos examined is mentioned as the *n* value. To minimize any image saturation bias, RNA in situ staining reactions were carefully monitored and stopped when the *dand5* domain was first evident.

### RNAseq

Raw reads were mapped to the zebrafish GRCz11 genome using STAR version 2.7.3a^59^ with the following non-default parameters: ╌alignEndsType ╌Local ╌outFilterMultimapNmax 1000 ╌seedSearchStartLmax 30 ╌sjdbScore 2–outMultimapperOrder Random. Genomic sequence indices for STAR were built using exon-junction coordinates from Ensembl r92^60^. Read counts per protein-coding gene were computed by summing the total number of reads overlapping the gene annotation by at least 10 nucleotides. All reads were used and contributed for 1/(number of mapping loci) to the gene counts. Per gene annotation was obtained by concatenating all Ensembl isoforms together. A total number of reads mapping to protein-coding genes and their lengths were used to normalize to RPKM (Reads Per Kilobase per Million mapped reads). For comparison, the average RPKMs of the following house-keeping genes were calculated: *actb1* (Actin, beta 1; cytoskeletal), *arpc2* (Actin related protein complex; cytoskeletal), *eif2a* (Eukaryotic translation initiation factor 2 A; translation), *ddx39b* (DEAD box polypeptide 39B; RNA splicing), *pabpn1* (Poly(A) binding protein, nuclear 1; RNA splicing), and *rps6* (Ribosomal protein S6; ribosomal protein). LabxDB^61^ was employed to manage sequencing samples.

### Immunofluorescence staining

For immunofluorescence staining, embryos were fixed in 4% PFA for 1 h at RT on a rocking platform, followed by 2 washes in 1× PBS^−^ for 15 min each. For staining of LRO explants, embryos were dissected using a scalpel into anterior and posterior halves. Posterior halves (LRO explants) were collected and transferred to a 24-well plate and washed twice for 15 min in PBST. LRO explants and whole embryos were blocked for 2 h at RT in CAS-Block diluted 1:10 in PBST. The blocking reagent was replaced by an antibody solution (anti-acetylated tubulin antibody, diluted 1:700 in CAS-Block; c2181 Sigma) and incubated overnight at 4 °C. In the morning, the antibody solution was removed and explants/embryos were washed twice for 15 min in PBS^−^. The secondary antibody (diluted 1:1000 in CAS-Block; c2181 Sigma) was added together with Phalloidin (1:200) and incubated for a minimum of 3 h at RT. Before photo documentation, embryos or explants were briefly washed in PBS^−^ and transferred onto a microscope slide.

### Western blot

Embryos were lysed with 10 µl/embryo RIPA buffer (radio immuno precipitation assay buffer) and centrifuged at maximum speed for 15 min at 4 °C and the supernatant was transferred into a new tube. The supernatant was boiled with 1× Laemmli Loading Buffer for 5 min at 95 °C. Probes were transferred to a 4–20% sodium dodecyl sulfate (SDS) gel (BIO RAD Mini-PROTEAN TGX Gels) and gel ran for 1 h at 120 V on a BIO RAD Mini-PROTEAN Tetra System. SDS Gel and nitrile-cellulose membrane were equilibrated in blotting buffer for 30 min and blotted for 1 h at 350 mA. The membrane was dissected and blocked (5% milk powder in Tbs_t_) for 1 h at room temperature. Incubation with primary antibodies (monoclonal anti-α-tubin produced in mouse, Sigma Aldrich T9026, 1:3000; monoclonal anti-Dicer1 produced in mouse, BioLegend MMS5130, 1:100) overnight at 4 °C. Membranes were washed in blocking solution (5% milk powder in Tbs_t_) and incubated with 2° antibody (Anti-Mouse IgG-peroxidase, Sigma Aldrich A9044, 1:80.000) for 3 h at room temperature. Antibody was removed and membranes were washed with Tbs_t_ and developed using Pierce ECL Western Blotting Substrate (ThermoScientific, #362109) and recorded with an exposure time of 600 µs. Uncropped blots can be found in the source data file.

### Luciferase assay

Luciferase reporter assays were carried out using the Promega Dual-Luciferase® Reporter Assay System. Animal cap tissue, derived from 10 embryos per treatment, was transferred into a 1.5 ml Eppendorf tube, and the 0.1× MBSH buffer was removed, leaving the tissue moistened. The tissue was lysed and homogenized in 100 µl 1× passive lysis buffer by pipetting the suspension up and down, followed by 15 min incubation at RT. The lysate was centrifuged for 2 min at 21,951 × g and the upper phase was transferred into a new tube. The lysate was re-centrifuged and two 25 µl aliquots (technical duplicates) of each sample were transferred into a 96-well plate. 75 µl 1× Luciferase assay substrate was added through the GloMax® Explorer System and luminescence was determined. This step was repeated with 75 µl 1× Stop and Glow reagents. To calculate the relative luciferase units (RLU [%]), the ratio between luciferase and Renilla values was calculated and correlated to the wt control, which was set to 100%. Each sample was measured twice to validate the technical aspect of testing. In order to be valid, the technical replicates should have almost identical values, which was true in all our experiments.

### Statistics and reproducibility

Statistical calculations of marker gene expression patterns and cilia distribution were performed using one-sided Pearson’s chi-square test in statistical R. Adjustments for multiple comparisons were done by Bonferroni (*pitx2* expression) or Bonferroni–Holm (*nodal1* and *dand5* expression) corrections. For the statistical calculation of ciliation, a Wilcoxon-Match-Pair test was used (statistical R-3.0.1). Statistical calculations of the luciferase assays were done with a one-sided student’s *t* test for two independent means in statistical R. Bonferroni corrections were implemented when multiple comparisons were conducted. At least three independent successful biological replicates (embryo batches) were used for each experimental setup. The source data file depicts all individual experiments/data points, mean values with standard deviations, and *p* values.

### Mouse strains

All mouse experiments were performed in accordance with guidelines of the RIKEN Center for Biosystems Dynamics Research (BDR) and under an institutional license (A2016-01-6). Mice were maintained in the animal facility of the RIKEN Center for BDR. Noto-Cre^ERT2^ mice were described in ref. ^62^, Dicer^flox^ mice in ref. ^63^ (JAX stock #006001). Expression of the Noto-Cre^ERT2^ transgene in embryos was induced by oral administration of tamoxifen (Sigma) in corn oil to pregnant mice at a dose of 5 mg both 24 and 12 h before the late headfold stage.

### WISH analysis in mouse

WISH was performed according to standard procedures with digoxigenin-labeled riboprobes specific for Nodal or Dand5 mRNA^64^.

### Reporting summary

Further information on research design is available in the Nature Research Reporting Summary linked to this article.

## Supplementary information

Supplementary Information

Reporting Summary

## Data Availability

The authors declare that the main data supporting the findings of this study are available within the article and its Supplementary Information files. Source data are provided with this paper.

## Source data

Source Data
