# Supplementary material for: Bicc1 and Dicer regulate left-right patterning through post-transcriptional control of the Nodal inhibitor Dand5
Source: Nat Commun. 2021 Sep 16;12:5482. doi: 10.1038/s41467-021-25464-z (PMC8446035; doi:10.1038/s41467-021-25464-z)
Supplement: Supplementary file 1 — Supplementary Information [file 41467_2021_25464_MOESM1_ESM.pdf]

Supplementary Materials

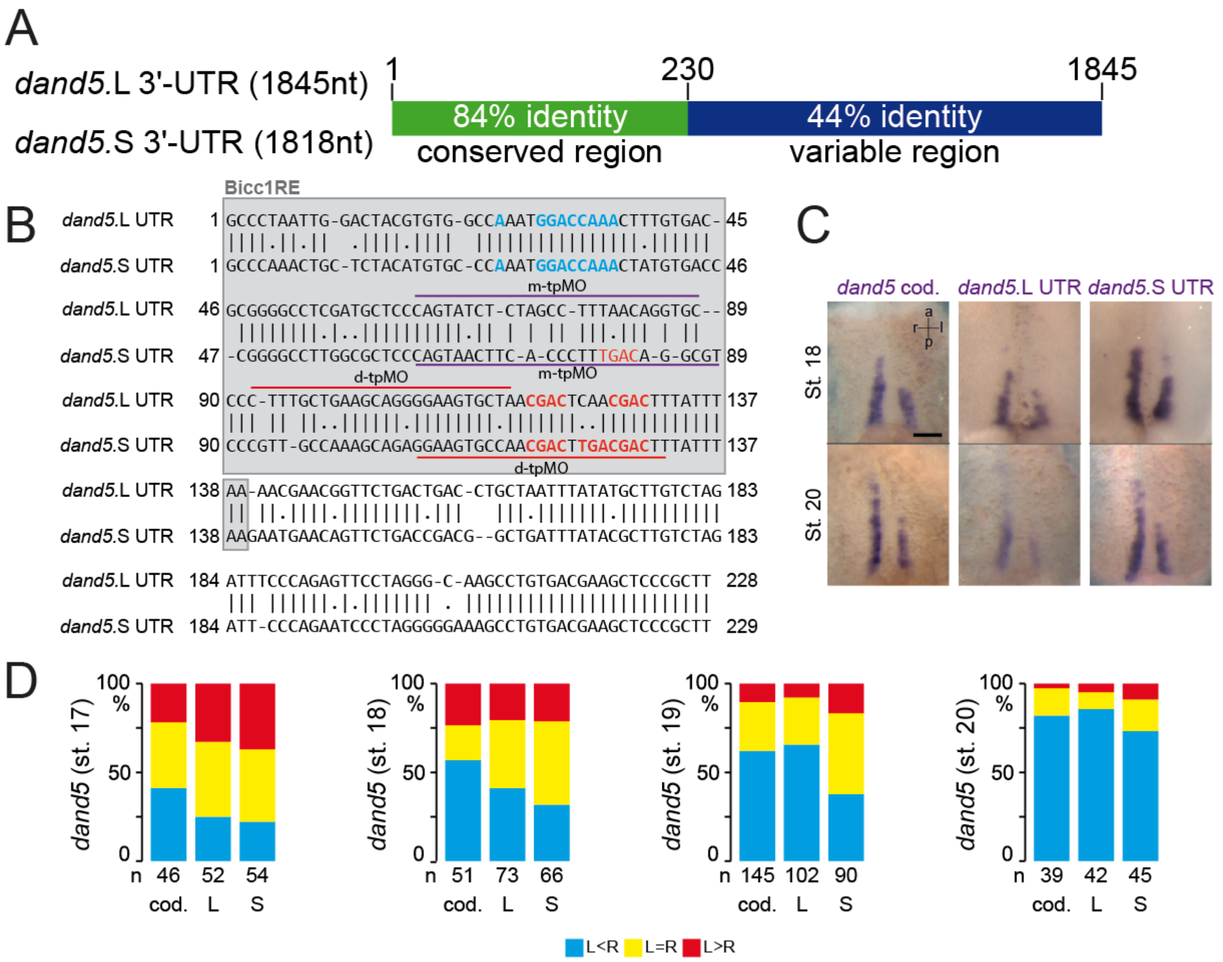

Supplementary Figure 1. Characterization of *dand5* 3'-UTRs

(A) Conservation of 3'-UTR sequences between S- and L-alloalles of *X. laevis*.

(B) Sequence alignment of the proximal 228 respective 229 nucleotides of *dand5* 3'-UTRs of S- and L-alloalleles. Minimal Bicc1 responsive element (Bicc1RE) is indicated by a grey background. The positions of the m-tpMOs and d-tpMOs are marked by blue and red lines, respectively. Two to three (S-UTR) potential Bicc1 binding motifs (based on [1]) are highlighted in red. Putative miR133 interaction site is indicated by blue letters.

(C) Representative dorsal explants of stage 18 (top row) and stage 20 (bottom row) embryos hybridized with antisense RNA probes specific for the *dand5* coding sequence (left), or the 3'-UTRs of *dand5* S- (middle) and L-allele (right).

(D) Quantification of results of a time course analysis from stage 17-20. Following *in situ* hybridization and visual judgement, scoring was carried out according to [2] i.e. bilateral symmetric (L=R), left stronger than right (L>R) or vice versa (L<R). n in the x-axis represent number of embryos analyzed.

Scale bar in (C) represents 100 µm. st., stage; a, anterior; l, left; r, right; p, posterior. Listing of individual experiments can be found in the source data file.

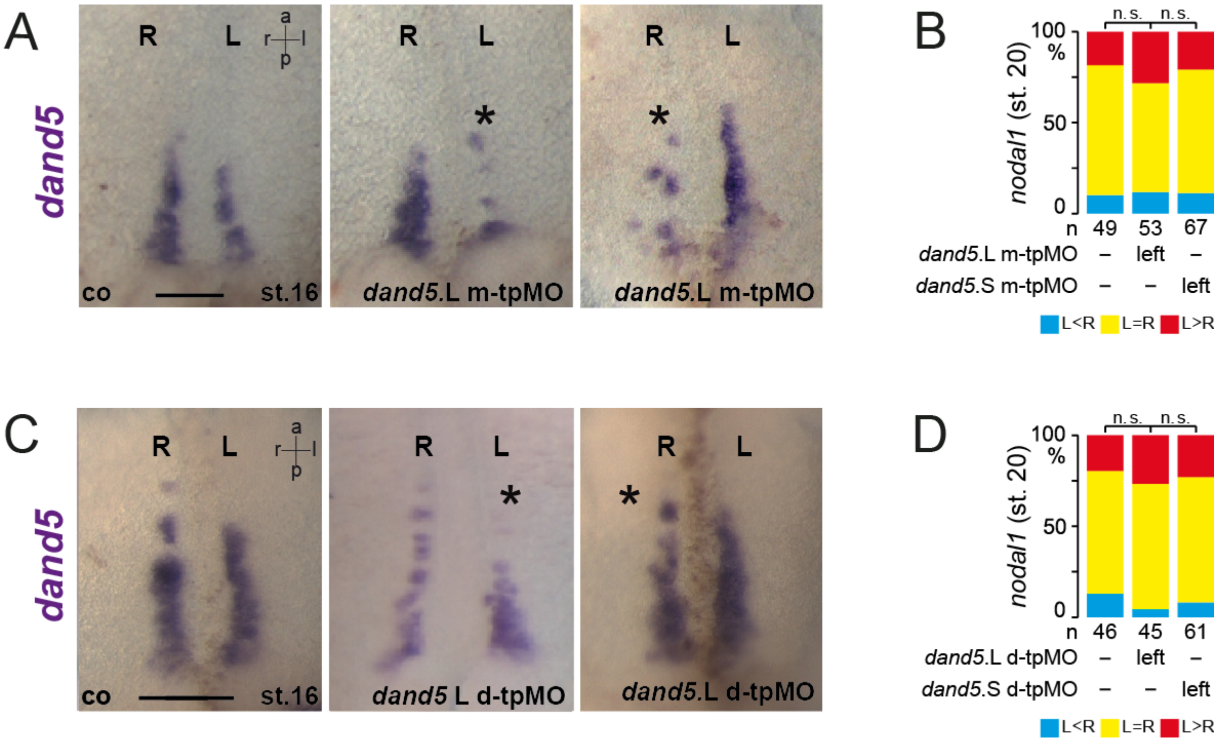

Supplementary Figure 2. *dand5* and *nodal1* expression in tpMO injected embryos.

(A) *dand5* expression in pre-flow stage 16 of controls, left- or right-sided m-tpMO injections.

Note that irrespective of which half was targeted, *dand5* mRNA was reduced.

(B) Quantification of *nodal1* expression at stage 20 of wt controls and left-sided m-tpMO injected embryos. No effect on *nodal1* mRNA by m-tpMO treatment.

(C) Wt *dand5* expression following injections of d-tpMO in pre-flow specimens (st. 16).

(D) Quantification of *nodal1* mRNA signals. Note no difference at stage 20 between wt controls and left-sided d-tpMO injections.

MO pmol/embryo: m-tpMO (L or S, 0.8); d-tpMO (L or S, 1). Asterisks in (A and C) mark injected side. Numbers (n) represent analyzed specimens from >3 independent experiments. st., stage; a, anterior; l, left; r, right; p, posterior; n.s., not significant. Scale bars in (A and C) represents 100 µm. p-values and listing of individual experiments can be found in the source data file. Statistical analyses were done with a one-sided Pearson's chi-square test (Boniferroni-Holm corrected).

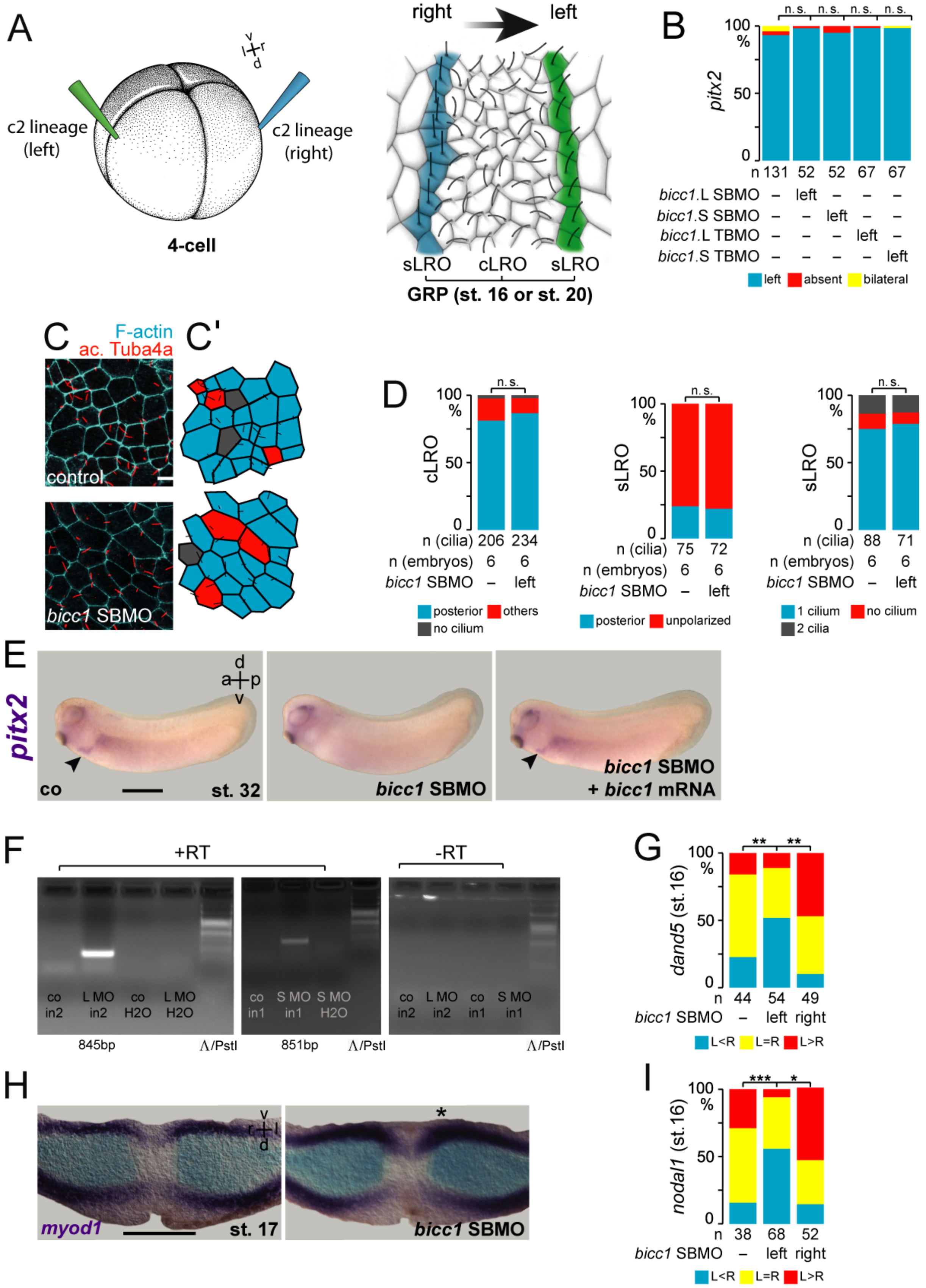
A
c2 lineage (left)
c2 lineage (right)
4-cell
right
left
sLRO
cLRO
sLRO
GRP (st. 16 or st. 20)
B
pitx2
n.s.
n 131 52 52 67 67
bicc1.L SBMO
bicc1.S SBMO
bicc1.L TBMO
bicc1.S TBMO
left
absent
bilateral
C
F-actin
ac. Tuba4a
C'
control
bicc1 SBMO
D
cLRO
n (cilia) 206 234
n (embryos) 6 6
posterior
others
no cilium
sLRO
n (cilia) 75 72
unpolarized
n (cilia) 88 71
1 cilium
2 cilia
E
pitx2
co
st. 32
bicc1 SBMO
bicc1 SBMO + bicc1 mRNA
F
+RT
-RT
co in2
L MO in2
co H2O
L MO H2O
co in1
S MO in1
S MO H2O
845bp
851bp
Λ/PstI
G
dand5 (st.16)
n 44 54 49
left
right
L<R
L=R
L>R
H
myod1
st. 17
I
nodal1 (st.16)
n 38 68 52

Supplementary Figure 3. Characterization of *bicc1* morphants

(A) Schematic depiction of injection scheme at the 4-cell stage to target specifically left (green) or right (blue) flow sensing cells at the gastrocoel roof plate (GRP; left-right organizer, LRO), which is shown as a dorsal explant of the archenteron at stage 19 Adapted from [3] and [4].

(B) Quantification of allele specific *bicc1* knockdowns. Note individual L or S MO (TB or SB; 1 pmol/embryo) has no effect on *pitx2* asymmetry.

(C) Unaltered LRO ciliation in *bicc1* morphants. Representative dorsal explants of control and *bicc1* morphant specimens are stained with anti- ac. tubulin (red) and phalloidin (F-actin, green). (C') Cartoon of the assessment of cilia polarization.

(D) Quantification of ciliary parameters in *bicc1* morphants (1 pmol). Note cilia polarization of flow generating cLRO and targeted flow sensing lateral sLRO cells was not affected by *bicc1* LoF. Ciliation of sLRO cells was not altered either.

(E) *pitx2* expression in representative control (co), *bicc1* morphant and specimen in which both SBMOs (L and S) and a full-length *bicc1* mRNA not targeted by the MOs were co-injected.

(F) RT-PCR on stage 18 embryos which were injected with *bicc1* SBMOs (L or S) showing intron retention (intron2, in2) or intron1, in1), respectively. No PCR products were found in negative control samples where reverse transcriptase was omitted (-RT).

(G, I) Quantification of *dand5* and *nodal1* expression in *bicc1* morphants at pre-flow stage 16. Both *dand5* and *nodal1* mRNA were strongly reduced by *bicc1* knockdown, irrespective of which side was targeted. (see Figure 2D,E).

(H) Expression of *myoD* at stage 17 showed that fate and morphogenesis of lateral sLRO was not affected by *bicc1* LoF.

MO pmol/embryo: *bicc1* SBMO (L and S, each 1); *bicc1* SBMO (L or S, 1). Numbers (n) in (B, D, G and I) represent analyzed specimens from >3 independent experiments. st., stage; a, anterior; l, left; r, right; p, posterior; d, dorsal; v, ventral; n.s., not significant; ***, very highly significant, $p < 0.001$; **, highly significant, $p < 0.01$. Asterisk in (H) marks injected side. Scale bars represent in (E) 1mm, in (H) 100 µm and in (C) 10 µm. p-values and listing of individual experiments can be found in the source data file. Statistical analyses were done with two-sided student t-test for 2 independent means (D, Boniferroni corrected) or with a one-sided Pearson's chi-square test (B, G and I, Boniferroni-Holm corrected).

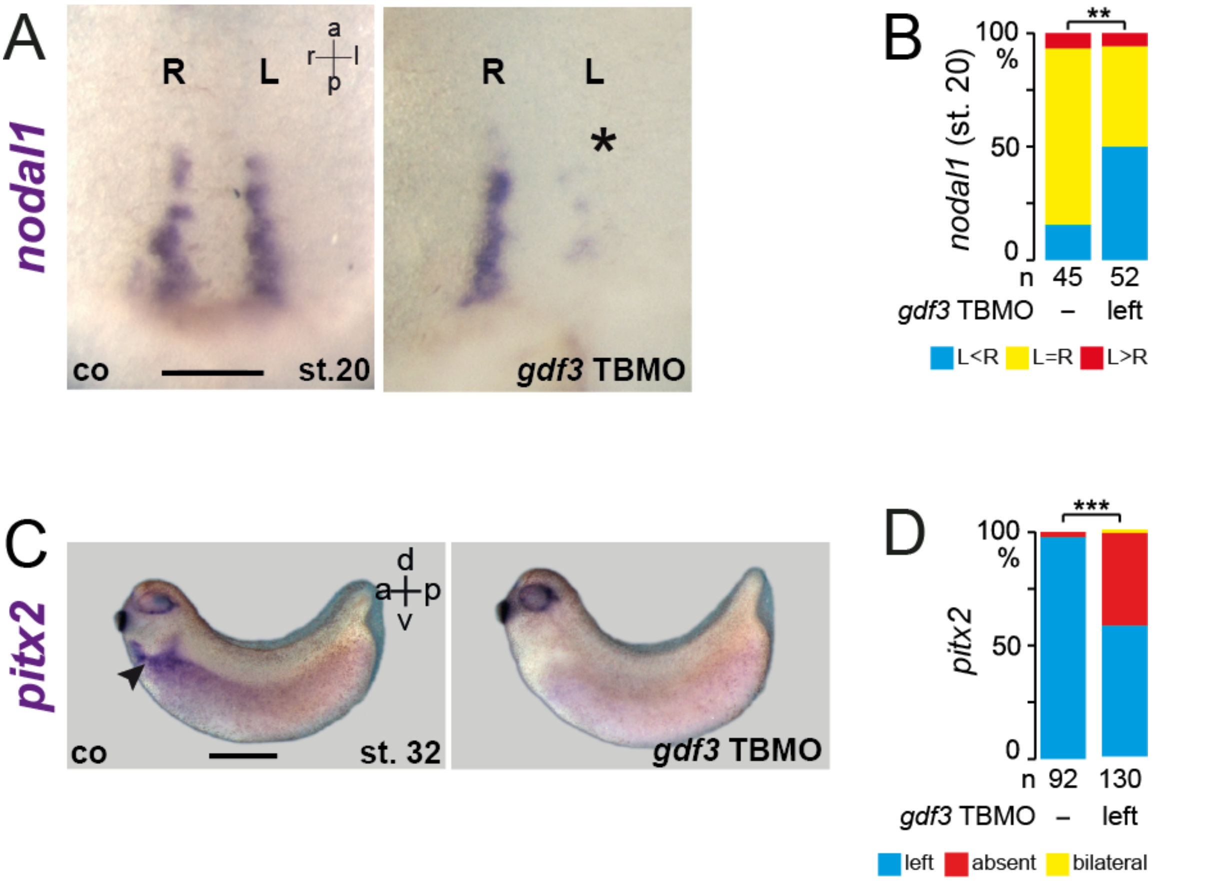

Supplementary Figure 4. *nodal1* transcription in sLRO cells requires Gdf3 signaling.

(A) *nodal1* sLRO expression at stage 20 in controls (co) and *gdf3* TBMO (0,3 pmol/embryo) unilaterally left injected specimens. Note reduced *nodal1* staining on the targeted side.

(B) Quantification of *nodal1* signals.

(C) *pitx2* asymmetry in the LPM was lost when *gdf3* MO was injected on the left.

(D) Quantification of *pitx2* expression in co and *gdf3* morphants.

Numbers (n) in (B and D) represent analyzed specimens from >3 independent experiments. Statistical analyses were done with a one-sided Pearson's chi-square test. n.s., not significant; ***, very highly significant, $p < 0.001$; **, highly significant, $p < 0.01$. p-values and listing of individual experiments can be found in the source data file.Asterisk in (A) marks injected side. Scale bars represent in (A) represents 100 µm and in (C) 1 mm. st., stage; a, anterior; l, left; r, right; p, posterior; d, dorsal; v, ventral;

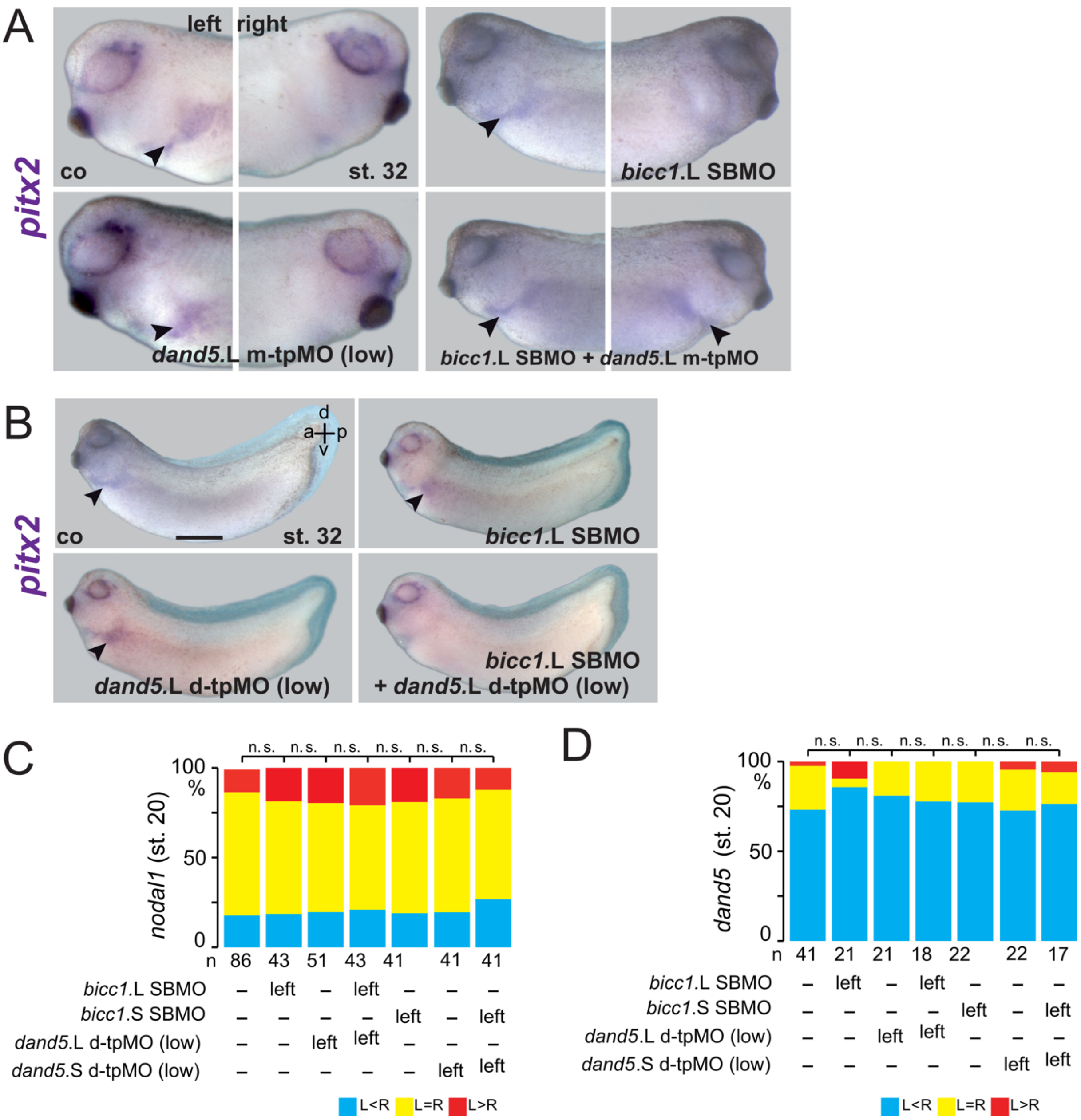

Supplementary Figure 5. ***pitx2*** **expression, but not** ***nodal1*** **transcription is altered by a combined treatment with low dose tpMO and single allele specific bicc1 knockdown.**

(A) Controls (co) as well as right sided injections of single L allele specific *bicc1* SBMO or low dose of m-tpMO have no effect. Combining both MOs induces ectopic *pitx2*.

(B) Injecting a mix of L allele specific *bicc1* SBMO and reduced d-tpMO concentrations into the left lineage results in loss of *pitx2* asymmetry. Individual MOs are ineffective.

(C) Quantification of *nodal1* mRNA expression at the LRO. Controls (co) did not differ from specimens which were injected with low dosages of d-tpMO (L or S) or allele specific *bicc1* SBMO (L or S; 1 pmol). This was also true for embryos where both MOs were co-injected.

(D) No effect on *dand5* asymmetry by left sided injections of low dosages of d-tpMO (L or S) or allele specific *bicc1* SBMO (L or S; 1 pmol) or in combination.

MO pmol/embryo: *bicc1* SBMO (L or S, 1); m-tpMO low (L or S, 0.4); d-tpMO low (L or S, 0,5). Scale bar in (B) represents 1 mm. Arrowheads in (A and B) point to *pitx2* positive LPMs. Numbers (n) in (C) and (D) represent analyzed specimens from ≥ 3 independent experiments. Statistical analyses were done with a one-sided Pearson's chi-square test (Boniferroni-Holm corrected). n.s., not significant; $p > 0.05$. p-values and listing of individual experiments can be found in the source data file. st., stage; a, anterior; p, posterior; d, dorsal; v, ventral; n.s., not significant.

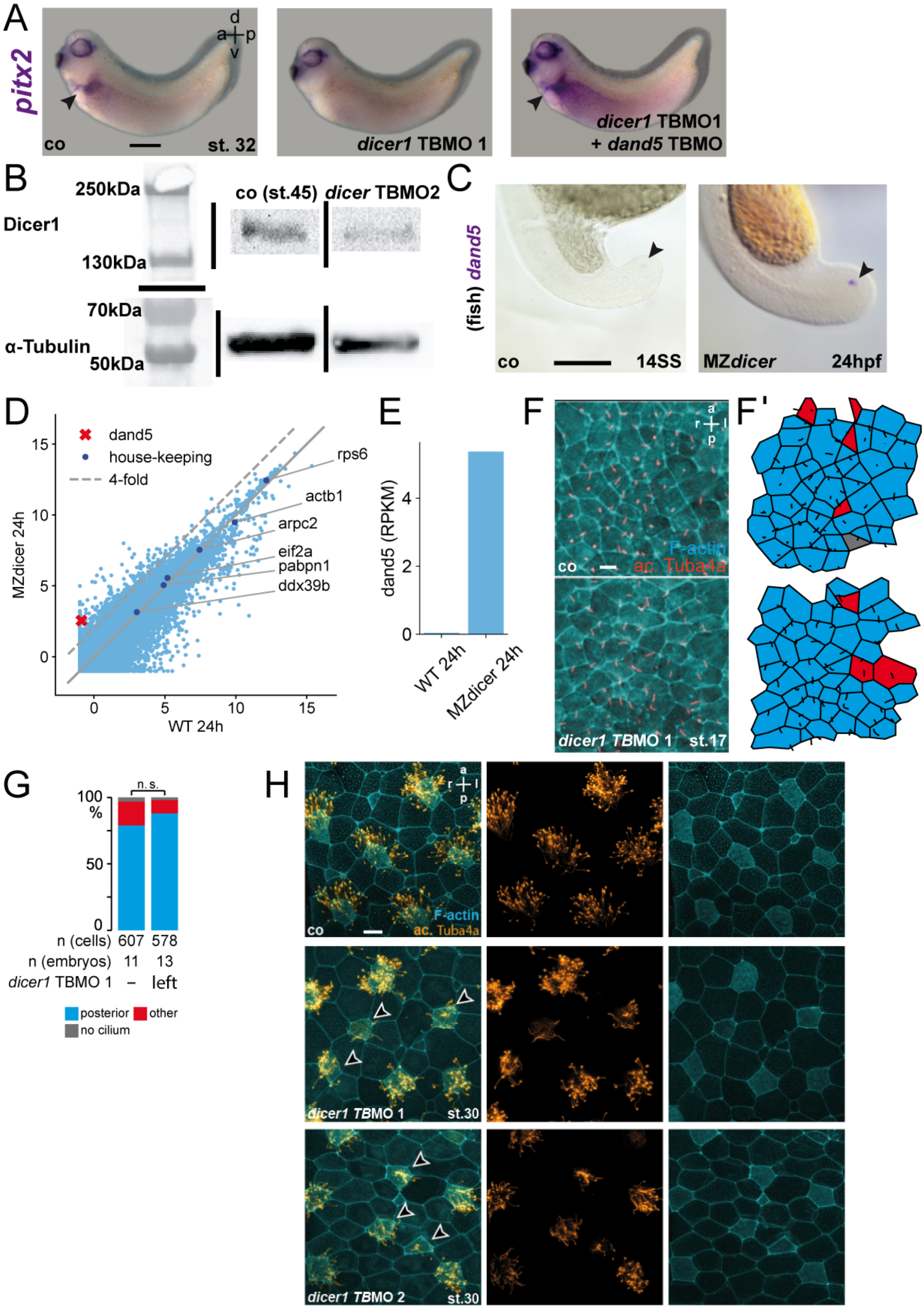

Supplementary Figure 6. ***dicer1*** **acts in post-flow stages during** ***Xenopus*** **LR axis formation.**

(A) Loss of *pitx2* expression in the left LPM (arrowhead) of st. 32 *dicer1* morphants was rescued by parallel knockdown of *dand5*.

(B) Western blot demonstrates *dicer1* TPMO2 (1 pmol) specificity. Proteins of controls and *dicer1* morphants at st. 45 were isolated and an anti-Dicer1 antibody was used for detection. Note strong signal reduction of Dicer1 in morphants. Anti-tubulin staining served as loading control.

(C) Sustained *dand5* expression in *dicer* mutant fish at 24hpf. Note *dand5* mRNA was basically absent in wt fish at 14 somite stage (SS), in contrast to 24hpf MZ*dicer* mutants. Arrowhead marks faint (co.) or strong (MZ*dicer*) *dand5* staining. Since KV was not specifically targeted, we cannot rule out indirect effects of *dicer* loss on *dand5* asymmetry.

(D) Scatter-plot comparing the expression of protein-coding genes in wt with their expression in MZ*dicer* zebrafish embryos at 24hpf (scale: log2 RPKM+0.5 on both axis). *Dand5* gene is marked by red cross. The dashed line represents a 4-fold increase. RPKM; Reads per kilo base per million mapped reads. Six housekeeping genes are highlighted and were not affected in dicer mutants.

(E) Bar-plot comparing *dand5* gene expression in wt (left) with its expression in MZ*dicer* zebrafish embryos at 24hpf. RPKM values wt: 0.040 and MZ*dicer*: 5.4.

(F) Wt LRO morphology and ciliation in *dicer1* morphants at st. 17 compared to un-injected control specimen, as shown by IF using an anti ac. Tuba4a antibody (red) and counterstaining of actin using phalloidin (blue). (F') Cartoon shows the assessment of cilia polarization.

(G) Quantification of cilia polarization in *dicer1* morphants. Statistical analyses were done with two-sided student t-test for 2 independent means. n represents number of cells or embryos which were analyzed.

(H) Dicer1 is required for ciliogenesis of epidermal multi-ciliated cells. Cilia and subcortical actin were stained by immunofluorescence (IF) using an antibody against acetylated tubulin (ac. Tuba4a; orange) and Phalloidin (blue) for F-actin. Compared to controls (co) cilia were substantially shortened upon *dicer1* TPMO1 or TPMO2 injection. Arrowheads point to targeted cells.

MO pmol/embryo: *dicer1* TBMO1 (1,5); *dicer1* TBMO2 (1); *dand5* TPMO (0,5). Scale bars in (A) represents 1 mm in (C) 200 µm and in (F, H) 10 µm. st., stage; a, anterior; l, left; r, right; p, posterior; d, dorsal; v, ventral; n.s., not significant. p-values and listing of individual experiments can be found in the source data file.

A

```
hsa-bicc1 3'UTR 109nt 5' ...GGGUGUCUGGUAUCA--GGACCAAA...
                                 |||||       ||||||||
        hsa-miR-133b  3'     AUCGACCAACUUCCCCUGGUUU
```

B

```
mmu-bicc1 3'UTR 261nt 5' ...GAGUGUCUGGUAUCA--GGACCAAA...
                                 |||||       ||||||||
     mmu-miR-133b-3p  3'     GUCGACCAACUUCCCCUGGUUU
```

C

```
xtr bicc1 3'UTR 115nt 5' ...AAGCCACGGUACCAA-GGACCAAA...
                                  |||       |||||||
        xtr-miR-133b  3'    AUCGACCAACUUCCCCUGGUU
```

Supplementary Figure 7. Conserved miR-133 binding sites in the *bicc1* 3'UTR.

Targetscan analysis [5,6] predicts miR133 hybridization to the human (A; hsa), mouse (B; mmu) and *Xenopus tropicalis* (C; xtr) *bicc1* 3'UTR. Nucleotide (nt) position of 3'UTR where the seed sequence starts is indicated.

Name	Sequence 5'– 3'	pmol/Embryo	Specificity	Origin
m-tpMO L	GCACCTGTTAAAGGCTAGAGATACT	High 0.8<br>Low 0.4		This work
m-tpMO S	ACGCCTGTCAAAGGGTGAAGTTACT	High 0.8<br>Low 0.4		This work
d-tpMO L	TAGCACTTCCCCTGCTTCAGCAAAG	High 1<br>Low 0.5	*dand5* MO rescue	This work
d-tpMO S	AAGTCGTCAAGTCGTTGGCACTTCC	High 1<br>Low 0.5	*dand5* MO rescue	This work
bicc1-SPMO1 L	GGGAATAGACTCACCCTGTAACATT	High 1<br>Low 0.5	RT PCR; *bicc1* mRNA / *dand5* MO rescue	This work
bicc1-SPMO2 S	CCCAACAAGCAAGCTCTTACCTTCT	High 1<br>Low 0.5	RT PCR; *bicc1* mRNA / *dand5* MO rescue	This work
bicc1 TBMO1 L	CCATTGTGCTACTGCCGCCGCTAAC	1		[7] (xBic-C-MO2)
bicc1 TBMO2 S	TAGACTCGCACTGAGCCGCCATTCT	1		[7] (xBic-C-MO1)
dand5-TBMO	CTGGTGGCCTGGAACAACAGCATGT	0.5		[8] (Coco1-MO)
gdf3-TBMO	CACAACTCTGCCATGTTGACTTCTC	0.3		[8] (derrière-MO)
dicer1-TBMO1	TGCAGGGCTTTCATAAATCCAGTGA	1.5		[9]
dicer1-TBMO2	CATGAGCTGAAGTCCTGCCATGC	1	Western Blot / *dand5* MO rescue	This work
pkd2 TBMO	GCCACTATCTCTTCAATCATCTCCG	1		[10]
zfPkd2 TBMO	AGGACGAACGCGACTGGAGCTCATC	1-4ng		[11]

Supplementary table 1. Morpholino oligomers used.

Primer name	Sequence 5'– 3'
dand5.L 3'-UTR forward primer	GCCCTAATTGGACTACGTGTGGCC
dand5.L 3'-UTR reverse primer	GCAGGACAATATAAAAACATGAGGC
gdf3 3'-UTR forward primer	ATAGATCTGTTTGCTTTGGAGATTGTTCTC
gdf3 3'-UTR reverse primer	ATAGATCTGGGTTAAATCACATTTATTTCC
bicc1.L forward primer	GCCACCCTTTCTCTTTACTAAACA
bicc1.L reverse primer	CTCTGCTTGGTTATTCCTGTTGGAGT
bicc1.S forward primer	GTGTTGAATTCTACTCACGAGGGAA
bicc1.S reverse primer	CTCGGCTTGGTTATTCCTATTGGAA

Supplementary table 2. PCR primers used.

References

1. Minegishi, K. *et al.* Fluid flow-induced left-right asymmetric decay of Dand5 mRNA in the mouse embryo requires Bicc1-Ccr4 RNA degradation complex. *Nature communications* (In Press).

2. Schweickert, A. *et al.* The nodal inhibitor Coco is a critical target of leftward flow in *Xenopus*. *Current Biology* **20,** 738–743; 10.1016/j.cub.2010.02.061 (2010).

3. Tisler, M., Schweickert, A. & Blum, M. *Xenopus*, an ideal model organism to study laterality in conjoined twins. *Genesis: the Journal of Genetics and Development* **55**, 55:e22993; https://doi.org/10.1002/dvg.22993 (2017)

4. Blum, M. & Ott, T. Animal left-right asymmetry. *Current Biology* **28,** 301-304; https://doi.org/10.1016/j.cub.2018.02.0735. Lewis, B. P., Burge, C. B. & Bartel, D. P. Conserved seed pairing, often flanked by adenosines, indicates that thousands of human genes are microRNA targets. *Cell* **120,** 15–20; 10.1016/j.cell.2004.12.035 (2005).

6. Friedman, R. C., Farh, K. K.-H., Burge, C. B. & Bartel, D. P. Most mammalian mRNAs are conserved targets of microRNAs. *Genome research* **19,** 92–105; 10.1101/gr.082701.108 (2009).

7. Maisonneuve, C. *et al.* Bicaudal C, a novel regulator of Dvl signaling abutting RNA-processing bodies, controls cilia orientation and leftward flow. *Development* **136,** 3019–3030; 10.1242/dev.038174 (2009).

8. Vonica, A. & Brivanlou, A. H. The left-right axis is regulated by the interplay of Coco, Xnr1 and derrière in *Xenopus* embryos. *Developmental biology* **303,** 281–294; 10.1016/j.ydbio.2006.09.039 (2007).

9. Agrawal, R., Tran, U. & Wessely, O. The miR-30 miRNA family regulates *Xenopus* pronephros development and targets the transcription factor Xlim1/Lhx1. *Development* **136,** 3927–3936; 10.1242/dev.037432 (2009).

10. Tran, U. *et al.* The RNA-binding protein bicaudal C regulates polycystin 2 in the kidney by antagonizing miR-17 activity. *Development* **137,** 1107–1116; 10.1242/dev.046045 (2010).

11. Schottenfeld, J., Sullivan-Brown, J. & Burdine, R. D. Zebrafish curly up encodes a Pkd2 ortholog that restricts left-side-specific expression of southpaw. *Development* **134,** 1605–1615; 10.1242/dev.02827 (2007).
