# Supplementary material for: Bicc1 and Dicer regulate left-right patterning through post-transcriptional control of the Nodal inhibitor Dand5
Source: Nat Commun. 2021 Sep 16;12:5482. doi: 10.1038/s41467-021-25464-z (PMC8446035; doi:10.1038/s41467-021-25464-z)
Supplement: Supplementary file 2 — Reporting Summary [file 41467_2021_25464_MOESM2_ESM.pdf]

nature research

Corresponding author(s): Axel Schweickert
Last updated by author(s): Sep 8, 2021

Reporting Summary

Nature Research wishes to improve the reproducibility of the work that we publish. This form provides structure for consistency and transparency in reporting. For further information on Nature Research policies, see our Editorial Policies and the Editorial Policy Checklist.

Statistics

For all statistical analyses, confirm that the following items are present in the figure legend, table legend, main text, or Methods section.

n/a	Confirmed	
☐	☒	The exact sample size (*n*) for each experimental group/condition, given as a discrete number and unit of measurement
☐	☒	A statement on whether measurements were taken from distinct samples or whether the same sample was measured repeatedly
☐	☒	The statistical test(s) used AND whether they are one- or two-sided<br>*Only common tests should be described solely by name; describe more complex techniques in the Methods section.*
☒	☐	A description of all covariates tested
☒	☐	A description of any assumptions or corrections, such as tests of normality and adjustment for multiple comparisons
☐	☒	A full description of the statistical parameters including central tendency (e.g. means) or other basic estimates (e.g. regression coefficient) AND variation (e.g. standard deviation) or associated estimates of uncertainty (e.g. confidence intervals)
☐	☒	For null hypothesis testing, the test statistic (e.g. *F*, *t*, *r*) with confidence intervals, effect sizes, degrees of freedom and *P* value noted<br>*Give P values as exact values whenever suitable.*
☒	☐	For Bayesian analysis, information on the choice of priors and Markov chain Monte Carlo settings
☒	☐	For hierarchical and complex designs, identification of the appropriate level for tests and full reporting of outcomes
☒	☐	Estimates of effect sizes (e.g. Cohen's *d*, Pearson's *r*), indicating how they were calculated

Our web collection on statistics for biologists contains articles on many of the points above.

Software and code

Policy information about availability of computer code

Data collection	No specific code was used.
Data analysis	STAR version 2.7.3a Mapping RNA reads to zebrafish genome GRCz11<br>Fiji/ImageJ 1.48i for image analysis<br>Acrobat Illustrator (version CS6) and Acrobat Photoshop (version CS6) for image processing<br>LabxDB was employed to manage sequencing samples.<br>statistical R R-3.0.1 by R Core Team statistical analysis

For manuscripts utilizing custom algorithms or software that are central to the research but not yet described in published literature, software must be made available to editors and reviewers. We strongly encourage code deposition in a community repository (e.g. GitHub). See the Nature Research guidelines for submitting code & software for further information.

Data

Policy information about availability of data

All manuscripts must include a data availability statement. This statement should provide the following information, where applicable:

- Accession codes, unique identifiers, or web links for publicly available datasets
- A list of figures that have associated raw data
- A description of any restrictions on data availability

The authors declare that the main data supporting the findings of this study are available within the article and its Supplementary Information files.

Field-specific reporting

Please select the one below that is the best fit for your research. If you are not sure, read the appropriate sections before making your selection.

- [x] Life sciences
- [ ] Behavioural & social sciences
- [ ] Ecological, evolutionary & environmental sciences

For a reference copy of the document with all sections, see nature.com/documents/nr-reporting-summary-flat.pdf

Life sciences study design

All studies must disclose on these points even when the disclosure is negative.

Sample size	No sample-size calculation was performed before the experiments and numbers are in the typical range for our modelorganisms.<br>The number of suitable frog and fish embryos and their survival is strongly connected to the fitness or heath status of the mother. Consequently, an excess of embryos are often used in the experiments and the number of analyzed embryos vary among each experiment. In addition, manipulations are often not fully penetrant in the Xenopus/fish system, which asks for higher sample sizes in experiments to have statistical sound result. Because the experimental outcome is not predictable in every case, the numbers of used embryos vary as well. Finally, clutches of frog and fish can contain hundrets to thousands embryos and thus the quantity is not limiting the experimenter in most cases and has therefore no impact on animal welfare issues.
Data exclusions	Experiments were excluded when untreated or mock-injected control embryos showed developmental defects in high frequency. This approach is generally accepted in the Xenopus field.
Replication	At least 3 independent experiments have been performed which used different embryo batches. All attempts at replication were successful.
Randomization	This method does not apply to our embryonic experimental setup in frog embryos. control embryos from the same clutch were used in each case.
Blinding	When experimental outcomes were scored in a subjective manner (i.e staining patterns of mRNA expression),<br>the obtained results were generally counterchecked in a blinded way. All other experiments were not blinded because data acquisition were obtained in a automated manner (Luciferase measurement, RNAseq).

Reporting for specific materials, systems and methods

We require information from authors about some types of materials, experimental systems and methods used in many studies. Here, indicate whether each material, system or method listed is relevant to your study. If you are not sure if a list item applies to your research, read the appropriate section before selecting a response.

Materials & experimental systems

n/a	Involved in the study	
[ ]	[x]	Antibodies
[x]	[ ]	Eukaryotic cell lines
[x]	[ ]	Palaeontology and archaeology
[ ]	[x]	Animals and other organisms
[x]	[ ]	Human research participants
[x]	[ ]	Clinical data
[x]	[ ]	Dual use research of concern

Methods

n/a	Involved in the study	
[x]	[ ]	ChIP-seq
[x]	[ ]	Flow cytometry
[x]	[ ]	MRI-based neuroimaging

Antibodies

Antibodies used	anti-acetylated tubulin antibody Sigma Aldrich, T7451; monoclonal anti-α-tubin, Sigma Aldrich T9026; monoclonal anti-Dicer, BioLegend MMS5130; Anti-Mouse IgG-peroxidase, Sigma Aldrich A904; anti mouse igG Sigma Aldrich C2181
Validation	Information on the supplier homepage for anti ac-tubulin AB: "Monoclonal Anti-α-Tubulin antibody produced in mouse has been used: In immunofluorescence Analysis; In western blotting/ Immunoblotting; For immunolabelling cells in electron microscopy References: Centrosomes split in the presence of impaired DNA integrity during mitosis. Hut HM Molecular and Cellular Biology 14 (5), 1993-2004, (2003); see also Piperno, G., and Fuller, M., J. Cell Biol., 101, 2085 (1985).<br>and LeDizet, M., and Piperno, G., Proc. Natl. Acad. Sci. USA, 84, 5720 (1987).<br><br>Sigma: Anti-Mouse IgG (whole molecule) F(ab')2 fragment–Cy3 antibody produced in sheep has been used:<br>• in immunolabeling of Hela cells[4]<br>• as secondary antibody in immunocytochemistry, immunofluorescence and immunohistochemistry.

Dicer AK for western Plot: VishlaghiN,etal.2020.FrontCellDevBiol.8:338.PubMed
Anti-Mouse IgG (whole molecule)–Peroxidase antibody has been used in western blotting , immunohistochemistry and protein pin array assay. Anti-Mouse IgG (whole molecule)–Peroxidase antibody may be used in direct ELISA

Animals and other organisms

Policy information about studies involving animals; ARRIVE guidelines recommended for reporting animal research

Laboratory animals	Xenopus laevis (males 5-6 years, female 5- 10 years); Mus musculus (males 5-10 months, female 3-6 months) ; zebrafish (males and females 5 to 18 months).
Wild animals	Wild animals were not used in this study.
Field-collected samples	In this study, samples were not collected in the field .
Ethics oversight	Animals were handled in accordance with German regulations (Tierschutzgesetz) and approved by the Regional Council Stuttgart (A379/12 Zo, 'Molekulare Embryologie', V340/17 ZO and V349/18 ZO, 'Xenopus Embryonen in der Forschung'). Zebrafish in in accordance with the Princeton University Institutional Animal Care and Use Committee (IACUC) guidelines. All mouse experiments were performed in accordance with guidelines of the RIKEN Center for Biosystems Dynamics Research (BDR) and under an institutional license (A2016-01-6).

Note that full information on the approval of the study protocol must also be provided in the manuscript.
