# Supplementary material for: Bicc1 and Dicer regulate left-right patterning through post-transcriptional control of the Nodal inhibitor Dand5
Source: Nat Commun. 2021 Sep 16;12:5482. doi: 10.1038/s41467-021-25464-z (PMC8446035; doi:10.1038/s41467-021-25464-z)
Supplement: Supplementary file 3 — Source Data [file 41467_2021_25464_MOESM3_ESM.pdf]

Figure 1B

					Mean	SD	Bonferroni corrections p values
	Luc+mBicc1/Luc For individual experiments>>				Mean	SD	
Dand5 L UTR Luciferase		0,64	0,64	0,34	0,54	0,173205	0,01
Dand5 S UTR Luciferase		0,77	0,61	0,28	0,553	0,249867	0,0364
	Luc+Bicc1/Luc For individual experiments>>				Mean	SD	
Dand5 L UTR Luciferase		0,1	0,12	0,18	0,13	0,041633	0,00001
Dand5 S UTR Luciferase		0,3	0,18	0,25	0,243	0,060277	0,00001

1D

											Bonferroni corrections p values
	Luc+Bicc1/Luc For individual experiments>>								Mean	SD	
Dand5 S UTR Luciferase	1-1818	0,2	0,09	0,15					0,146	0,041793	0,00001
	1-568	0,28	0,31	0,22					0,27	0,045826	0,00001
	568-1818	0,83	0,92	0,9					0,88	0,052915	0,0322
	1-103	0,9	1,1	0,88					0,956	0,125033	n.s
	1-139	0,12	0,089	0,17					0,117	0,037995	0,00001
tdgf1 (positive control)		0,26	0,08	0,16	0,158	0,23	0,18	0,27	0,226	0,045092	0,00001
cyclin B1 (negative control)		1,1	1	0,94	0,89	0,95	0,99	1,07	0,982	0,090323	n.s

1F

						Bonferroni corrections p values
Dand5 S UTR Luciferase	uc+/Luc For individual experiments>>			Mean	SD	
bicc1 mRNA	0,298	0,28	0,235	0,271	0,03245	0,00001
dand5 S m-tpMO	0,51852	0,600433	0,406879	0,508611	0,097157	0,0021
dand5 S d-tpMO + bicc1 mRNA	4,111	3,76	3,367	3,746	0,372198	0,00004
dand5 S d-tpMO	3,95	3,21	3,342	3,500667	0,394691	0,00001
dand5 L UTR Luciferase	uc+/Luc For individual experiments>>			Mean	SD	
bicc1 mRNA	0,415281	0,51546	0,649089	0,52661	0,180046	0,0036
dand5 L m-tpMO	0,310802	0,333534	0,35353	0,332622	0,021379	0,00001
dand5 L d-tpMO + bicc1 mRNA	3,513321	1,691856	1,323881	2,176353	0,172376	0,00001
dand5 L d-tpMO	3,864681	1,257159	1,591279	2,237706	0,418871	0,00001

2H

dand5 probe

	L<R	L=R	L>R
co (st.20)	20	5	
dand5 L d-tpMO (left injection)	11	4	
dand5 S d-tpMO (left injection)	11	3	1
co (st.20)	6	7	1
dand5 L d-tpMO (left injection)	7	5	1
dand5 S d-tpMO (left injection)	9	11	3
co (st.20)	8	6	1
dand5 L d-tpMO (left injection)	8	4	1
dand5 S d-tpMO (left injection)	5	4	2
co (st.20)	22	6	
dand5 L d-tpMO right injection)	20	4	1
co (st.20)	9	7	1
dand5 L d-tpMO right injection)	8	5	1
co (st.20)	12	20	5
dand5 L d-tpMO right injection)	8	19	4
co (st.20)	24	6	1
dand5 S d-tpMO right injection)	17	5	2
co (st.20)	17	13	1
dand5 S d-tpMO right injection)	15	15	2
co (st.20)	15	10	3
dand5 S d-tpMO right injection)	17	14	3

	L<R	L=R	L>R	n	Bonferroni-Holm corrections p-values
co (st.20)	133	80	13	226	
dand5 L d-tpMO (left injection)	26	13	2	41	1.
dand5 L d-tpMO right injection)	36	28	6	70	1.
dand5 S d-tpMO (left injection)	25	18	6	49	1.
dand5 S d-tpMO right injection)	49	34	7	90	1.

2I

dand5 probe

	L<R	L=R	L>R
co st.16	6	9	3
dand5 L d-tpMO (left injection)	7	12	1
dand5 L d-tpMO (right injection)	8	20	3
co st.16	2	14	1
dand5 L d-tpMO (left injection)	4	25	1
dand5 L d-tpMO (right injection)	4	23	1
co st.16	2	12	2
dand5 L d-tpMO (left injection)	2	8	2
dand5 L d-tpMO (right injection)	3	6	2
co st.16	4	27	2
dand5 L d-tpMO (left injection)	4	24	4
dand5 L d-tpMO (right injection)	7	25	5
co st.16	5	16	1
dand5 S d-tpMO (left injection)	6	22	1
dand5 S d-tpMO (right injection)	6	25	
co st.16	6	24	4
dand5 S d-tpMO (left injection)	2	25	7
dand5 S d-tpMO (right injection)	9	23	2
co st.16	6	20	3
dand5 S d-tpMO (left injection)	5	20	6
dand5 S d-tpMO (right injection)	9	23	2

	L<R	L=R	L>R	n	Bonferroni-Holm corrections p-values
co (st.16)	31	122	16	169	
dand5 L d-tpMO (left injection)	17	69	8	94	1.
dand5 L d-tpMO (right injection)	22	74	11	107	1.
dand5 S d-tpMO (left injection)	13	67	14	94	1.
dand5 S d-tpMO (right injection)	24	71	4	99	1.

3C *dand5* probe

	R>L	R=L	R<L
co (st.20)	17	4	1
bicc1 SBMO (left injection)	12	7	
bicc1 SBMO (right injected)	5	3	6
co (st.20)	8	5	
bicc1 SBMO (left injection)	11		
bicc1 SBMO (right injected)	7	6	2
co (st.20)	13	4	1
bicc1 SBMO (left injection)	17	6	
bicc1 SBMO (right injected)	2	15	6
co (st.20)	43	12	9
bicc1 SBMO (left injection)	22	4	1
bicc1 SBMO + bicc1 mRNA (left injection)	24	12	18
co (st.20)	13	13	3
bicc1 SBMO (left injection)	36	8	5
bicc1 SBMO + bicc1 mRNA (left injection)	15	7	11
co (st.20)	19	10	4
bicc1 SBMO (left injection)	15	0	3
bicc1 SBMO + bicc1 mRNA (left injection)	15	10	10
co (st.20)	44	22	14
bicc1 mRNA (left injection)	21	17	21
bicc1 mRNA (right injection)	45	10	5
co (st.20)	51	6	15
bicc1 mRNA (left injection)	39	11	19
bicc1 mRNA (right injection)	12	2	1
co (st.20)	16	8	6
bicc1 mRNA (left injection)	9	15	8
bicc1 mRNA (right injection)	15	4	1

	R>L	R=L	R<L	n	Bonferroni-Holm corrections p-values	
co (st.20)	224	84	53	361		
bicc1 SBMO (left injection)	113	25	9	147	0,034	0,00001
bicc1 SBMO + bicc1 mRNA (left injection)	54	29	39	122	0,0046	
bicc1 SBMO (right injected)	14	24	14	52	0	
bicc1 mRNA (left injection)	69	43	48	160	0,0117	
bicc1 mRNA (right injection)	72	16	7	95	0,034	

3D *nodal1* probe

	R>L	R=L	R<L
co (st.20)	4	7	2
bicc1 SBMO (left injection)	13	5	1
bicc1 SBMO (right injected)	1	7	5
co (st.20)	6	10	4
bicc1 SBMO (left injection)	15	7	2
bicc1 SBMO + bicc1 mRNA (left injection)	4	12	4
bicc1 SBMO (right injected)	1	12	8
co (st.20)	3	6	1
bicc1 SBMO (left injection)	11	5	1
bicc1 SBMO + bicc1 mRNA (left injection)	5	4	6
bicc1 SBMO (right injected)	2	19	9
co (st.20)		43	
bicc1 mRNA (left injection)	2	43	
bicc1 mRNA (right injection)		29	1
co (st.20)	8	24	10
bicc1 mRNA (left injection)	7	18	6
bicc1 mRNA (right injection)	6	16	9
co (st.20)	7	20	8
bicc1 mRNA (left injection)	6	15	5
bicc1 mRNA (right injection)	6	13	9
co (st.20)	6	12	4
bicc1 SBMO (left injection)	13	7	1
bicc1 SBMO + bicc1 mRNA (left injection)	4	5	2

	R>L	R=L	R<L	n	Bonferroni-Holm corrections p-values	
co (st.20)	34	122	29	185		
bicc1 SBMO (left injection)	52	24	5	81	.0057	
bicc1 SBMO + bicc1 mRNA (left injection)	13	21	12	46	1.	0,000537
bicc1 SBMO (right injected)	4	38	22	64	0,0094	
bicc1 mRNA (left injection)	15	76	11	102	1.	
bicc1 mRNA (right injection)	12	58	19	89	1.	

Figure 4A

					Mean	SD	p-values compared to control
	Luc+Bicc1/Luc For individual experiments>>						
derriere S UTR Luciferase	0,385039	0,15526	0,32563		0,288643	0,113	0,000248

4C *gdf3* probe

	R>L	R=L	R<L
co (st.20)	4	4	3
bicc1 SBMO (left injection)	3	4	2
bicc1 SBMO (right injection)	2	4	2
co (st.20)	1	7	2
bicc1 SBMO (left injection)	2	7	1
bicc1 SBMO (right injection)	2	6	1
co (st.20)	6	4	1
bicc1 SBMO (left injection)	5	9	4
bicc1 SBMO (right injection)	4	8	1
co (st.20)	2	13	1
bicc1 SBMO (left injection)	4	13	3
bicc1 SBMO (right injection)	1	23	1
co (st.20)	2	16	2
bicc1 SBMO (left injection)	2	18	3
bicc1 SBMO (right injection)	4	17	4
co (st.20)	2	10	1
bicc1 SBMO (left injection)	3	10	3
bicc1 SBMO (right injection)	6	19	5

	R>L	R=L	R<L	n	Bonferroni-Holm corrections p-values
co (st.20)	17	54	10	81	
bicc1 SBMO (left injection)	19	61	16	96	1.
bicc1 SBMO (right injection)	19	77	14	110	1.

4E *nodal1* probe

	R>L	R=L	R<L
co (st.20)	4	9	3
bicc1 SBMO (left injection)	6	5	1
bicc1 SBMO + gdf3 mRNA (left injection)	4	26	2
co (st.20)	3	8	3
bicc1 SBMO (left injection)	7	7	
bicc1 SBMO + gdf3 mRNA (left injection)	4	10	1
co (st.20)	6	12	4
bicc1 SBMO (left injection)	12	9	
bicc1 SBMO + gdf3 mRNA (left injection)	3	5	3
co (st.20)	5	12	14
bicc1 SBMO (left injection)	14	10	3
bicc1 SBMO + gdf3 mRNA (left injection)	6	10	16

	R>L	R=L	R<L	n	Bonferroni-Holm corrections p-values
co (st.20)	18	41	24	83	
bicc1 SBMO (left injection)	39	31	4	74	0
bicc1 SBMO + gdf3 mRNA (left injection)	17	51	22	90	0.631

4G *pitx2* probe

	wt	bilateral	absent	right
co	26			
bicc1 SBMO (left injection)	13	2	13	
bicc1 SBMO + derriere mRNA (left injection)	28		4	
co	33			
bicc1 SBMO (left injection)	10		21	
bicc1 SBMO + derriere mRNA (left injection)	27		3	
co	32			
bicc1 SBMO (left injection)	21		8	
bicc1 SBMO + derriere mRNA (left injection)	27		1	
co	27	1		
bicc1 SBMO (left injection)	16		12	
bicc1 SBMO + derriere mRNA (left injection)	25	1	5	

	wt	bilateral	absent	n	Bonferroni corrections p-values	
co	118	1	0	119		
bicc1 SBMO	60	2	54	116	0	0,00001
bicc1 SBMO + derriere mRNA	107	1	13	121	0,0089	

Figure 5A — *pitx2* probe

	wt	absent	bi	right
co	48			
bicc1 L SBMO (right injection)	28			
dand5 L m-tpMO (right injection)	30			
dand5 L m-tpMO + bicc1 L SBMO (right injection)	22		4	5
co	31	1		1
bicc1 S SBMO (right injection)	31		2	2
dand5 S m-tpMO (right injection)	30		3	
dand5 S m-tpMO + bicc1 S SBMO (right injection)	15	1	11	
co	28	1		
bicc1 L SBMO (right injection)	25		1	1
dand5 L m-tpMO (right injection)	18	1	1	1
dand5 L m-tpMO + bicc1 L SBMO (right injection)	10	2	14	2
bicc1 S SBMO (right injection)	19		1	
dand5 S m-tpMO (right injection)	23	1	1	
dand5 S m-tpMO + bicc1 S SBMO (right injection)	6	1	9	
co	29			
bicc1 L SBMO (right injection)	31		2	
dand5 L m-tpMO (right injection)	19			
dand5 L m-tpMO + bicc1 L SBMO (right injection)	20		8	
co	29			
bicc1 S SBMO (right injection)	32			
dand5 S m-tpMO (right injection)	36		2	
dand5 S m-tpMO + bicc1 S SBMO (right injection)	23		5	2

	wt	absent	bi	right	n	Bonferroni corrections p-values		
co	165	2	0	1	168			
bicc1 L SBMO (right injection)	84	0	3	1	88	1.		
dand5 L m-tpMO (right injection)	67	1	1	1	70	1.	0,00001	0,00001
dand5 L m-tpMO + bicc1 L SBMO (right injection)	52	2	26	7	87	0,0001		
bicc1 S SBMO (right injection)	82	0	3	2	87	1.		
dand5 S m-tpMO (right injection)	89	1	6	0	96	1.	0,00001	0,00001
dand5 S m-tpMO + bicc1 S SBMO (right injection)	44	2	25	2	73	0,0001		

5C — *dand5* probe

	L<R	L=R	L>R
co (st.20)	12	6	2
bicc1 S SBMO (right injection)	12	8	3
dand5 S m-tpMO (right injection)	7	8	5
dand5 S m-tpMO + bicc1 S SBMO (right injection)	5	8	13
co (st.20)	20	2	
bicc1 S SBMO (right injection)	21	4	
dand5 S m-tpMO (right injection)	13	6	1
dand5 S m-tpMO + bicc1 S SBMO (right injection)	6	6	11
co (st.20)	15	10	1
bicc1 L SBMO (right injection)	5	10	
dand5 L m-tpMO (right injection)	6	7	2
dand5 L m-tpMO + bicc1 L SBMO (right injection)		9	6
co (st.20)	11	7	1
bicc1 L SBMO (right injection)	6	5	1
dand5 L m-tpMO (right injection)	7	6	2
dand5 L m-tpMO + bicc1 L SBMO (right injection)		4	5
co (st.20)	18	6	
bicc1 L SBMO (right injection)	10	8	2
dand5 L m-tpMO (right injection)	9	11	2
dand5 L m-tpMO + bicc1 L SBMO (right injection)	6	10	4
co (st.20)	13	5	2
bicc1 S SBMO (right injection)	10	4	2
dand5 S m-tpMO (right injection)	9	5	1
dand5 S m-tpMO + bicc1 S SBMO (right injection)	5	6	4

	L<R	L=R	L>R	n	Bonferroni-Holm corrections p-values		
co (st.20)	89	36	6	131			
bicc1 S SBMO (right injection)	43	16	5	64	0,8044		
dand5 S m-tpMO (right injection)	29	19	7	55	0,246	0,00001	0,00001
dand5 S m-tpMO + bicc1 S SBMO (right injection)	16	20	28	64	0,0001		
bicc1 L SBMO (right injection)	21	23	3	47	0,246		
dand5 L m-tpMO (right injection)	22	24	6	52	0,1145	0,00001	0,00001
dand5 L m-tpMO + bicc1 L SBMO (right injection)	6	23	15	44	0,0001		

5D — *pitx2* probe

	wt	absent	bi	right
co	27	1		
bicc1 S SBMO	22	4		1
dand5 S d-tpMO	26	1	1	
dand5 S d-tpMO + bicc1 S SBMO	13	18		
co	24	1		
bicc1 S SBMO	26	4		
dand5 S d-tpMO	20	1		
dand5 S d-tpMO + bicc1 S SBMO	10	15		
co	20		1	
bicc1 S SBMO	19	1		1
dand5 S d-tpMO	19	1	2	
dand5 S d-tpMO + bicc1 S SBMO	11	7	2	
co	31		1	
bicc1 S SBMO	24		1	
dand5 S d-tpMO	29		1	
dand5 S d-tpMO + bicc1 S SBMO	24	5	1	
co	16		1	
bicc1 L SBMO	26		1	1
dand5 L d-tpMO	27	4		
dand5 L d-tpMO + bicc1 L SBMO	10	6		1
co	26		1	
bicc1 L SBMO	21	1	1	
dand5 L d-tpMO	17	1	1	
dand5 L d-tpMO + bicc1 L SBMO	12	8	1	1
co	38	1	1	
bicc1 L SBMO	28	1	1	
dand5 L d-tpMO	30	1	2	
dand5 L d-tpMO + bicc1 L SBMO	13	12	2	2

	wt	absent	bi	right	n	Bonferroni corrections p-values		
co	182	3	5	0	190			
bicc1 S SBMO	91	9	1	2	103	0,7471		
dand5 S d-tpMO	94	3	4	0	101	1.	0,00001	0,00001
dand5 S d-tpMO + bicc1 S SBMO	58	45	3	0	106	0,0001		
bicc1 L SBMO	75	2	3	1	81	1.		
dand5 L d-tpMO	74	6	3	0	83	1.	0,00001	0,00001
dand5 L d-tpMO + bicc1 L SBMO	35	26	3	4	68	0,0001		

6E — *dand5* probe

	L<R	L=R	L>R
co (st.20)	15	7	0
dicer1 TBMO 1 (left injection)	5	4	7
co (st.20)	20	2	2
dicer1 TBMO 1 (left injection)	11	10	2
co (st.20)	38	0	1
dicer1 TBMO 1 (left injection)	64	15	6
co (st.20)	3	5	0
dicer1 TBMO 1 (left injection)	2	6	8
co (st.20)	23	4	1
dicer1 TBMO 1 (left injection)	67	58	33
co (st.20)	21	16	3
dicer1 TBMO 1 (left injection)	2	8	2
co (st.20)	10	3	1
dicer1 TBMO 1 (left injection)	12	7	5
co (st.20)	22	2	0
dicer1 TBMO 1 (left injection)	17	4	3
co (st.20)	13	5	7
dicer1 TBMO 1 (left injection)	4	4	12
co (st.20)	24	1	5
dicer1 TBMO 2 (left injection)	14	17	10
co (st.20)	21	3	4
dicer1 TBMO 2 (left injection)	12	10	6
co (st.20)	25	2	6
dicer1 TBMO 2 (left injection)	14	13	10

	L<R	L=R	L>R	n	Bonferroni-Holm corrections p-values
co (st.20)	235	50	30	315	
dicer1 TBMO 1 (left injection)	184	116	78	378	0,0001
dicer1 TBMO 2 (left injection)	40	40	26	106	0,0001

6F — *Dand5* probe

**Mouse 8.0**	R>L	R=L	R<L
WT	8		
Dicer CKO		3	

6G — *dand5* probe

**Zebrafish**	L<R	L=R	L>R	n	p-values compared to control
WT	15	5		20	
Mz*dicer*	5	13	1	18	0,0059669

6I — *pitx2* probe

	wt	absent	bilateral	right
co	13			
bicc1 S SBMO (left injection)	18	1		
dicer1 TBMO 1 (left injection)	17			
bicc1 S SBMO + dicer1 TBMO 1 (left injection)	5	13		
co	12			
bicc1 S SBMO (left injection)	19	1		
dicer1 TBMO 1 (left injection)	20	2		
bicc1 S SBMO + dicer1 TBMO 1 (left injection)	6	14		
co	30	1		
bicc1 L SBMO (left injection)	13			
dicer1 TBMO 1 (left injection)	12	4	1	
bicc1 L SBMO + dicer1 TBMO 1 (left injection)	3	11	1	
co	31	2		
bicc1 L SBMO (left injection)	13	1		2
dicer1 TBMO 1 (left injection)	14	6	2	2
bicc1 L SBMO + dicer1 TBMO 1 (left injection)	4	14	3	

	wt	absent	bilateral	right	n	Bonferroni corrections p-values				
co	86	3	0	0	89					
bicc1 S SBMO (left injection)	37	2	0	0	39	1.				
dicer1 TBMO 1 (left injection)	63	12	3	0	80	0,026	0,00001		0,00001	
bicc1 S SBMO + dicer1 TBMO 1 (left injection)	11	27	0	2	38	0				0,00001
bicc1 L SBMO (left injection)	26	1	0	0	29	1.		0,00001		
bicc1 L SBMO + dicer1 TBMO 1 (left injection)	7	25	4	2	36	0				
				0						

Figure 7A		*dand5* probe		
**Zebrafish**	L>R	L=R	L<R	n
WT	1	1	5	7
heterozygous	1	2	5	8
null mutant		4		4

7B		*dand5* probe			p-values compared to control
**Zebrafish**					
	L>R	L=R	L<R	n	
WT	1	17	30	48	
pkd2 MO	8	25	6	39	1,4552E-05

7C						Bonferroni corrections p values
Dand5 S UTR Luciferase	Luc+/Luc For individual experiments>>			Mean	SD	
bicc1 mRNA	0,1523	0,23112	0,2166	0,20000667	0,04194821	0,0001
bicc1 mRNA (low)	0,4452458	0,33784789	0,48967	0,42425456	0,07805743	0,0006
bicc1 mRNA (low) + pkd2 mRNA	0,0752231	0,2200858	0,1666	0,15396964	0,07325261	0,0001
bicc1 mRNA (low) + pkd2 TBMO	0,90912924	0,56591475	0,7789	0,764648	0,19200437	0,2033
pkd2 mRNA	7,6974	1,329	2,363636	3,7966787	3,4175047	0,0001
pkd2 TBMO	0,53727393	0,47828019	0,30085578	0,4388033	0,12305366	0,0042

Supplementary Figure 1D

dand5 coding probe

Stage	R>L	R=L	L>R
17	1	3	2
17	6	4	1
17	5	5	3
17	7	5	4
18	7	1	2
18	6	3	1
18	6	1	
18	5	3	5
18	5	2	4
19	7	4	1
19	17	5	2
19	10	4	2
19	10	3	2
19	11	6	2
19	20	10	3
19	15	8	3
20	9	1	
20	10	2	
20	8	2	
20	5	1	1

dand5 S UTR probe

Stage	R>L	R=L	L>R
17	1		5
17	4	13	6
17	1	1	6
17	3	4	3
17	3	4	
18	10	6	4
18	3	3	2
18	2	6	0
18	4	8	4
18	1	4	1
18	1	4	3
19	0	5	4
19	5	9	1
19	2	1	1
19	9	9	3
19	10	9	4
19	8	8	2
20	8	4	2
20	7	1	1
20	8	2	
20	7	1	0
20	3		1

dand5 L UTR probe

Stage	R>L	R=L	L>R
17		3	3
17	4	8	5
17	2	3	1
17	4	5	2
17	3	3	6
18	7	8	1
18	1	7	2
18	2	0	2
18	12	8	1
18	6	5	1
18	2	0	8
19	10	0	1
19	2	2	2
19	5	1	0
19	9	5	1
19	10	5	1
19	12	6	2
19	8	2	
19	11	6	1
20	3	0	0
20	12	1	1
20	6	1	
20	8	1	1
20	7	1	

dand5 coding probe

	R>L	R=L	L>R
st.17	19	17	10
st.18	29	10	12
st. 19	90	40	15
st. 20	32	6	1

dand5 S UTR probe

	R>L	R=L	L>R
st.17	12	22	20
st.18	21	31	14
st.19	34	41	15
st.20	33	8	4

dand5 L UTR probe

	R>L	R=L	L>R
st.17	13	22	17
st.18	30	28	15
st.19	67	27	8
st.20	36	4	2

Supplementary Figure 2B *nodal1* probe

	L<R	L=R	L>R
co (st.20)	1	9	4
dand5 L m-tpMO (right injection)	2	8	9
dand5 S m-tpMO (right injection)	2	6	5
co (st.20)	3	15	4
dand5 L m-tpMO (right injection)	4	10	6
dand5 S m-tpMO (right injection)	2	18	4
co (st.20)	1	11	1
dand5 L m-tpMO (right injection)	2	22	4
dand5 S m-tpMO (right injection)	2	12	2

	L<R	L=R	L>R	n	Bonferroni-Holm corrections p-values
co (st.20)	5	35	9	49	
dand5 L m-tpMO (right injection)	8	40	19	67	0,7937
dand5 S m-tpMO (right injection)	6	36	11	53	0,7937

2D *nodal1* probe

	L<R	L=R	L>R
co (st.20)	1	11	1
dand5 S d-tpMO (left injection)	2	23	5
dand5 L d-tpMO (left injection)		12	2
co (st.20)	2	9	3
dand5 S d-tpMO (left injection)	1	6	4
dand5 L d-tpMO (left injection)		5	4
co (st.20)	3	11	5
dand5 S d-tpMO (left injection)	2	13	5
dand5 L d-tpMO (left injection)	2	14	6

	L<R	L=R	L>R	n	Bonferroni-Holm corrections p-values
co (st.20)	6	31	9	46	
dand5 S d-tpMO (left injection)	5	42	14	61	0,6882
dand5 L d-tpMO (left injection)	2	31	12	45	0,597

Supplementary Figure 3B *pitx2* probe

	wt	bilateral	absent
co	27	2	
bicc1 L TBMO (left injection)	26		
bicc1 S TBMO (left injection)	20	1	
co	18	1	1
bicc1 L TBMO (left injection)	20		1
bicc1 S TBMO (left injection)	27		
co	19	1	
bicc1 L TBMO (left injection)	20		
bicc1 S TBMO (left injection)	19		
co	26	1	2
bicc1 L SBMO (left injection)	18		1
bicc1 S SBMO (left injection)	18		2
co	21	1	
bicc1 L SBMO (left injection)	17	1	1
bicc1 S SBMO (left injection)	16		1
co	20		
bicc1 L SBMO (left injection)	14		
bicc1 S SBMO (left injection)	15		

	wt	bilateral	absent	n	Bonferroni corrections p-values
co	64	4	1	69	
bicc1 L TBMO	66	0	1	67	1.
bicc1 S TBMO	66	1	0	67	1.
bicc1 L SBMO	49	1	2	52	1.
bicc1 S SBMO	49	0	3	52	1.

3D

LRO cells

wt	posterior	no cilium	others
Embryo 1	36		3
Embryo2	26	1	3
Embryo 3	32		7
Embryo 4	28	1	8
Embryo 5	23	1	7
Embryo 6	24	2	6

bicc1 SBMO (left injection)	posterior	no cilium	others
Embryo 1	29		3
Embryo2	34	3	3
Embryo 3	32	1	6
Embryo 4	38		3
Embryo 5	40		7
Embryo 6	30	1	4

	posterior	no cilium	others	n	p-values compared to control
wt	169	5	34	208	
bicc1 SBMO (left injection)	203	5	26	234	0,265277

somitic LRO cells

wt	posterior	unpolarized
Embryo 1	3	12
Embryo 2	5	14
Embryo 3	4	14
Embryo 4	4	11
Embryo 5	2	6

bicc1 SBMO (left injec	posterior	unpolarized
Embryo 1	3	13
Embryo 2	4	12
Embryo 3	4	12
Embryo 4	2	7
Embryo 5	3	12

	posterior	unpolarized	n	p-values compared to control
wt	18	57	75	
bicc1 SBMO (left injection)	16	56	72	0,798308

wt	1 cilium	no cilium	2 cilia
Embryo 1	13	2	2
Embryo 2	11	1	3
Embryo 3	20	3	2
Embryo 4	14	1	4
Embryo 5	8	3	1

bicc1 SBMO (left injec	1 cilium	no cilium	2 cilia
Embryo 1	14	2	2
Embryo 2	12	2	2
Embryo 3	9	1	2
Embryo 4	13	0	1
Embryo 5	8	1	2

	1 cilium	no cilium	2 cilia	n	p-values compared to control
wt	66	10	12	88	
bicc1 SBMO (left injection)	56	6	9	71	0,804281

3G *dand5* probe

	R>L	R=L	R<L
co st.16	2	9	2
bicc1 SBMO (left injected) st.16	8	9	1
bicc1 SBMO (right injected) st.16	1	3	9
	R>L	R=L	R<L
co st.16	2	9	
bicc1 SBMO (left injected) st.16	10	4	1
bicc1 SBMO (right injected) st.16	1	4	7
	R>L	R=L	R<L
co st.16	6	9	5
bicc1 SBMO (left injected) st.16	10	7	4
bicc1 SBMO (right injected) st.16	3	14	7

dand5	R>L	R=L	R<L	n	Bonferroni-Holm corrections p-values
co (st.16)	10	27	7	44	
bicc1 SBMO (left injected) st.16	28	20	6	54	0,0098
bicc1 SBMO (right injected) st.16	5	21	23	49	0,0094

3I *nodal1* probe

	R>L	R=L	R<L
co st.16	2	4	4
bicc1 SBMO (left injected) st.16	12	11	2
bicc1 SBMO (right injected) st.16	3	2	8
	R>L	R=L	R<L
co st.16	3	11	6
bicc1 SBMO (left injected) st.16	14	9	2
bicc1 SBMO (right injected) st.16	2	11	10
	R>L	R=L	R<L
co st.16	1	6	1
bicc1 SBMO (left injected) st.16	12	6	
bicc1 SBMO (right injected) st.16		5	11

nodal1	R>L	R=L	R<L	n	Bonferroni-Holm corrections p-values
co (st.16)	6	21	11	38	
bicc1 SBMO (left injected) st.16	38	26	4	68	0,0001
bicc1 SBMO (right injected) st.16	5	18	29	52	0,0327

Supplementary Figure 4B — *nodal1* probe

	R>L	R=L	R<L
co (st.20)	1	6	3
gdf3 TBMO (left injection)	8	3	2
co (st.20)	2	11	
gdf3 TBMO (left injection)	9	4	1
co (st.20)	4	18	
gdf3 TBMO (left injection)	9	16	

	R>L	R=L	R<L	n	p-values compared to control
co (st.20)	7	35	3	45	
gdf3 TBMO (left injection)	26	23	3	52	0,001515
	4,66666667	23,3333333	2		
	15	13,2692308	1,73076923		

4D — *pitx2* probe

	wt	bilateral	absent	right
co	31			
gdf3 TBMO (left injection)	26		13	
co	33		2	
gdf3 TBMO (left injection)	31	2	30	
co	26			
gdf3 TBMO (left injection)	18		10	

	wt	bilateral	absent	n	p-values compared to control
co	90	0	2	92	0,000029
gdf3 TBMO (left injection)	75	2	53	130	

Supplementary Figure 5C — *nodal1* probe

	L<R	L=R	L>R
co (st.20)	2	13	
bicc1 S SBMO (left injection)	3	12	1
dand5 S d-tpMO (left injection)	3	9	1
dand5 S d-tpMO + bicc1 S SBMO (left injection)	2	11	

	L<R	L=R	L>R
co (st.20)	4	8	3
bicc1 L SBMO (left injection)	2	9	4
dand5 L d-tpMO (left injection)	5	9	5
dand5 L d-tpMO + bicc1 L SBMO (left injection)	4	13	5

	L<R	L=R	L>R
co (st.20)	6	12	3
bicc1 L SBMO (left injection)	4	14	3
dand5 L d-tpMO (left injection)	4	15	4
dand5 L d-tpMO + bicc1 L SBMO (left injection)	4	7	2

	L<R	L=R	L>R
co (st.20)	1	5	2
bicc1 L SBMO (left injection)	2	4	2
dand5 L d-tpMO (left injection)	2	4	1
dand5 L d-tpMO + bicc1 L SBMO (left injection)	2	12	1

	L<R	L=R	L>R
co (st.20)		5	2
bicc1 S SBMO (left injection)	1	7	2
dand5 S d-tpMO (left injection)		8	3
dand5 S d-tpMO + bicc1 S SBMO (left injection)		6	2

	L<R	L=R	L>R
co (st.20)	2	17	1
bicc1 S SBMO (left injection)	4	7	4
dand5 S d-tpMO (left injection)	5	9	3
dand5 S d-tpMO + bicc1 S SBMO (left injection)	9	8	3

	L<R	L=R	L>R	n	Bonferroni-Holm corrections p-values
co (st.20)	16	59	11	86	
bicc1 L SBMO (left injection)	8	27	8	43	1.
dand5 L d-tpMO (left injection)	11	36	10	57	1.
dand5 L d-tpMO + bicc1 L SBMO (left injection)	9	25	9	43	1.
bicc1 S SBMO (left injection)	8	26	7	41	0,5972
dand5 S d-tpMO (left injection)	8	26	7	41	0,5972
dand5 S d-tpMO + bicc1 S SBMO (left injection)	11	25	5	41	0,3988

5D — *dand5* probe

	L<R	L=R	L>R
co (st.20)	14	6	1
bicc1 S SBMO (left injection)	17	5	
dand5 S d-tpMO (left injection)	16	5	1
dand5 S d-tpMO + bicc1 S SBMO (left injection)	13	3	1

	L<R	L=R	L>R
co (st.20)	16	4	
bicc1 L SBMO (left injection)	18	1	2
dand5 L d-tpMO (left injection)	17	4	
dand5 L d-tpMO + bicc1 L SBMO (left injection)	14	4	

	L<R	L=R	L>R	n	Bonferroni-Holm corrections p-values
co (st.20)	30	10	1	41	
bicc1 L SBMO (left injection)	18	1	2	21	1.
dand5 L d-tpMO (left injection)	17	4	0	21	1.
dand5 L d-tpMO + bicc1 L SBMO (left injection)	14	4	0	18	1.
bicc1 S SBMO (left injection)	17	5	0	22	1.
dand5 S d-tpMO (left injection)	16	5	1	22	1.
dand5 S d-tpMO + bicc1 S SBMO (left injection)	13	3	1	17	1.

Supplementary Figure 6C *dand5* probe

Zebrafish

	absent	faint present	present	n
WT (14ss)	13	3		16
WT (18ss)	10			10
MZdicer	5		13	18

	absent	faint present	present	n	p-values compared to control
WT (14ss)	13	3		16	
MZdicer	5		13	18	5,81E-05

	absent	faint present	present	n	
WT (18ss)	10			10	
MZdicer	5		13	18	0,000241

6G

WT	posterior	no cilium	others
embryo1	48	4	11
embryo2	45	3	7
embryo3	92	5	21
embryo4	43	0	9
embryo5	35	0	10
embryo6	31	2	5
embryo7	43	2	6
embryo8	39	0	12
embryo9	36	1	4
embryo10	36	2	9
embryo11	36	2	8

dicer TBMO 1 (left injection)	posterior	no cilium	others
embryo 1	39	4	4
embryo 2	42	1	7
embryo 3	31	1	6
embryo 4	49	2	3
embryo 5	35	1	5
embryo 6	46	3	4
embryo 7	37	0	3
embryo 8	27	2	6
embryo 9	31	0	1
embryo 10	19	1	4
embryo 11	55	0	1
embryo 12	48	3	9
embryo 13	47	0	1

	posterior	no cilium	others	n	p-values compared to control
WT	484	21	102	607	
dicer TBMO 1 (left injection)	506	18	54	578	0,000615

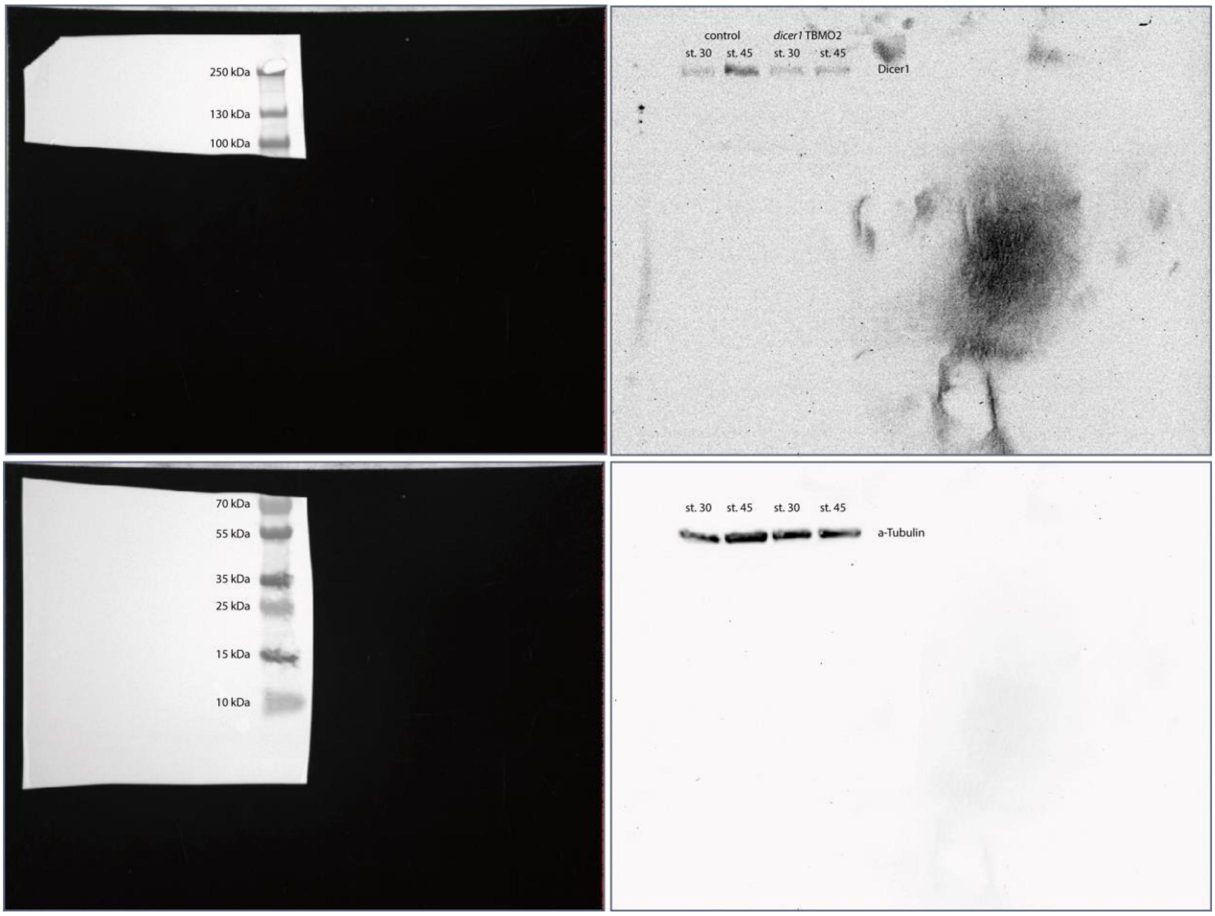

Source data to supplementary figure 6: Western blot was cut (after running) for photo documentation purposes. stage, st.
